# Rapid reprogramming of epigenetic and transcriptional profiles in mammalian culture systems

**DOI:** 10.1186/s13059-014-0576-y

**Published:** 2015-02-04

**Authors:** Colm E Nestor, Raffaele Ottaviano, Diana Reinhardt, Hazel A Cruickshanks, Heidi K Mjoseng, Rhoanne C McPherson, Antonio Lentini, John P Thomson, Donncha S Dunican, Sari Pennings, Stephen M Anderton, Mikael Benson, Richard R Meehan

**Affiliations:** Centre for Individualised Medicine, Faculty of Health Sciences, Linköping University, Linköping, 581 83 Sweden; MRC Human Genetics Unit, Institute of Genetics and Molecular Medicine, Western General Hospital, Crewe Road, Edinburgh, EH4 2XU UK; Centre for Cardiovascular Science, Queen’s Medical Research Institute, University of Edinburgh, 47 Little France Crescent, Edinburgh, EH16 4TJ UK; MRC Centre for Inflammation Research, Centre for Multiple Sclerosis Research and Centre for Immunity Infection and Evolution, University of Edinburgh, Edinburgh, EH16 4TJ UK

## Abstract

**Background:**

The DNA methylation profiles of mammalian cell lines differ from those of the primary tissues from which they were derived, exhibiting increasing divergence from the *in vivo* methylation profile with extended time in culture. Few studies have directly examined the initial epigenetic and transcriptional consequences of adaptation of primary mammalian cells to culture, and the potential mechanisms through which this epigenetic dysregulation occurs is unknown.

**Results:**

We demonstrate that adaptation of mouse embryonic fibroblasts to cell culture results in a rapid reprogramming of epigenetic and transcriptional states. We observed global 5-hydroxymethylcytosine (5hmC) erasure within three days of culture initiation. Loss of genic 5hmC was independent of global 5-methylcytosine (5mC) levels and could be partially rescued by addition of vitamin C. Significantly, 5hmC loss was not linked to concomitant changes in transcription. Discrete promoter-specific gains of 5mC were also observed within seven days of culture initiation. Against this background of global 5hmC loss we identified a handful of developmentally important genes that maintained their 5hmC profile in culture, including the imprinted loci *Gnas* and *H19*. Similar outcomes were identified in the adaption of CD4^+^ T cells to culture.

**Conclusions:**

We report a dramatic and novel consequence of adaptation of mammalian cells to culture in which global loss of 5hmC occurs, suggesting rapid concomitant loss of methylcytosine dioxygenase activity. The observed epigenetic and transcriptional re-programming occurs much earlier than previously assumed, and has significant implications for the use of cell lines as faithful mimics of *in vivo* epigenetic and physiological processes.

**Electronic supplementary material:**

The online version of this article (doi:10.1186/s13059-014-0576-y) contains supplementary material, which is available to authorized users.

## Background

DNA methylation in mammals involves attachment of a methyl group to a cytosine, usually in the context of a CpG dinucleotide, by a member of the DNA methyltransferase (DNMT) family of enzymes to yield 5-methylcytosine (5mC) [1]. The ability to establish and maintain DNA methylation patterns is essential for normal development in mammals [2]. The discovery of substantial amounts of 5-hydroxymethylcytosine (5hmC) in many mammalian tissues together with the identification of the ten-eleven translocation enzymes (TET1/2/3) that catalyze the conversion of 5mC to 5hmC have proved key to revealing a potential mechanism of ‘active’ (enzymatic) DNA demethylation in mammals. Currently, a ‘sequential oxidation’ model of active DNA demethylation is proposed, whereby a TET enzyme sequentially oxidizes 5hmC to 5-formylcytosine (5fC) and/or 5-carboxylcytosine (5caC) [3]; the latter two modified bases can be removed by the mammalian thymine DNA glycosylase and subsequently repaired to yield unmodified cytosine [4]. Alternatively, as DNMT1, a maintenance DNMT, does not recognize and re-methylate hemi-hydroxymethylated DNA generated during DNA replication, 5hmC may also be lost by replication-dependent dilution [5,6]. Interestingly, in some tissues, UHRF1 may direct DNMT1 to sites of hemi-hydroxymethylated DNA, thereby enabling maintenance of 5hmC patterns through cell division, although the evidence for this mechanism is currently inconclusive [1,7-9]. The discovery of TET/5hmC-mediated DNA demethylation pathways has transformed the view of DNA methylation in mammals from an inflexible and permanent repressive mark to that of a potentially dynamic, responsive and reversible process [6,8].

DNA methylation at a set of CpG island (CGI) promoters in transformed cell lines differs from that in the parental tissue of origin, exemplified by an increase in CGI methylation with immortalization and passage number [7,10-12]. Such *de novo* hypermethylation events are far less common in non-transformed primary cell lines, suggesting that such changes are associated with escape from replicative senescence in immortalized cells [10,13]. In a seminal study, Meissner and colleagues [14] used massively parallel reduced-representation bisulfite sequencing to show that astrocytes derived from *in vivo* neural progenitor cells displayed significantly less *de novo* promoter methylation than those derived *in vitro* from embryonic stem (ES) cells, and that the number of hypermethylated CGI promoters increased with passage number. Stable methylation at promoters also distinguished epiblast stem cells cultured *ex vivo* from early passage *in vivo*-derived epiblasts. Altered patterns of DNA methylation are established within a few days during *in vitro* outgrowth of the epiblast [15]. The origin of these culture-induced DNA methylation changes is unknown. *De novo* methylation events in culture may result from increased activity or inappropriate targeting of the methyltransferases DNMT3A/B or from loss of a DNA demethylase activity. In agreement with their proposed ‘demethylation’ role, mutations in *TET1* and *TET2*, which are frequent in many cancers, are associated with a hypermethylated promoter phenotype (as seen in acute myeloid leukemia), as are ‘*de novo*’ mutations in the *IDH1/2* genes that result in the production of 2-hydroxyglutarate instead of α-ketoglutarate, a competitive inhibitor of TET enzyme activity [6]. Over 20 years ago, Antequera, Boyes and Bird suggested that the observed *de novo* methylation of CGI promoters in transformed cells may result from loss of a demethylase activity in culture [11].

We have previously noted that many transformed cell lines possess substantially less absolute 5hmC levels compared with their tissues of origin [16]. Similar changes in global 5mC levels have not been observed [17-20]. As the relative levels of global 5hmC in cell lines do not correlate with those observed in the normal tissues of origin, we hypothesized that the low levels of global 5hmC in transformed cell lines reflected loss of a TET-associated methylcytosine dioxygenase activity upon adaptation of cells to culture. Here, we investigated the effect of culture adaptation on the methylome, hydroxymethylome and transcriptome of mouse embryonic fibroblasts (MEFs). We present evidence of rapid and genome-wide epigenetic and transcriptional remodeling of mammalian cell states in culture. Our results reveal an underappreciated difference between the epigenetic character of mammalian tissues and primary cell lines derived therefrom, and raise potential reservations about the use of such systems as faithful models of *in vivo* DNA methylation dynamics.

## Results

### Rapid, global loss of 5hmC, but not 5mC, occurs during adaptation of mammalian cells to culture

**Figure 1:**
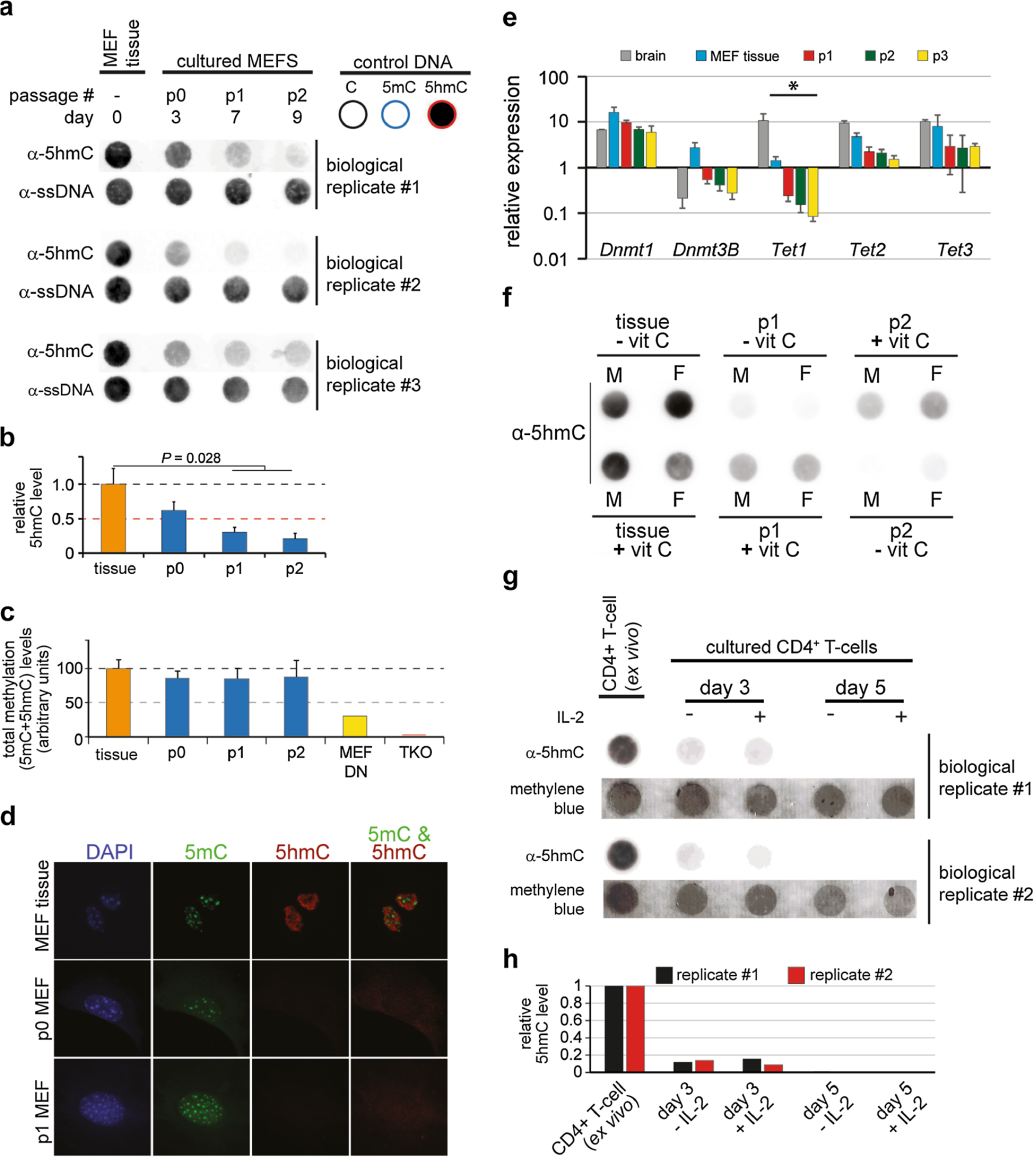
**Adaptation of mammalian cells to standard culture conditions results in 5hmC loss. (a)** Dot-blot of genomic DNA from primary MEF tissue and cultured cells derived therefrom with a 5hmC-specific antibody (500 ng were loaded in all lanes). ssDNA, single-stranded DNA. **(b)** Quantification of global 5hmC levels by densitometric analysis of dot-blots (±SE). P, passage. **(c)** Quantification of total (5hmC + 5mC) modification levels in DNA by LUMA. DNA from triple knock-out (*dnmt3a* ^-*/*-^, *dnmt*3b^-*/*-^, *dnmt*1^-*/*-^) mouse ES cells (TKO) which contain <2% DNA methylation was used as a negative control and was set to a value of 0 and DNA methylation levels in MEF tissue were set to 100. *Dnmt1* ^-*/*-^ MEF DNA (MEF DN ), which contains approximately 20% of normal methylation levels, is also shown (±SE). **(d)**. Fluorescence microscopy images of MEFs during adaptation to culture stained with antibodies against 5mC (green) and 5hmC (red). DNA was counterstained with DAPI (blue). The merged 5mC/5hmC image is shown on the right. **(e)** Bar-chart of quantitative RT-PCR showing reduced expression of *Tet1* upon adaptation to culture. Values are the average of three biological replicates normalized to *Gapdh* expression ± standard deviation. *P < 0.05, Mann-Whitney U-test. **(f)** Dot-blot of DNA (1 μg) from male and female primary MEFs showing partial rescue of global 5hmC levels by addition of vitamin C (1,000 μM). Top row shows partial recovery of global 5hmC after addition of vitamin C at passage 2. Bottom row shows reduction of 5hmC loss after addition of vitamin C at passage 1. **(g)** Dot-blot of DNA (500 ng) from naïve CD4^+^ T cells and cultured cells derived therefrom with a 5hmC-specific antibody. Two replicate experiments are shown. **(h)** Quantification of global 5hmC levels by densitometric analysis of dot-blots shown in (g). Values were normalized to a methylene blue loading control.

To determine the consequences of adaptation to culture on the mammalian methylome we purified genomic DNAs from primary MEFs (embryonic day (E)13.5 post coitum) and subsequently derived cultured MEFs at various time-points (3, 7 and 9 days after isolation). Each experiment consisted of MEF cells pooled from three to five embryos of mixed gender. The relative global 5hmC content of each sample was then determined by immuno dot-blot [16]. A rapid and progressive loss of 5hmC was observed upon adaptation of MEFs to standard culture conditions (Figure 1a). Quantification of spot intensity by densitometric analysis of four biological replicates revealed a 60% loss of global 5hmC by passage 1 (7 to 9 days in culture; *P* < 0.05; Figure 1b). In contrast, quantification of total modified cytosine (5mC + 5hmC) levels by luminometric methylation analysis (LUMA) revealed a small (4 to 8%) decrease in total modified cytosine in cultured cells (Figure 1c). The modest reduction in total modified cytosine is entirely consistent with 1) the substantial loss of 5hmC observed, as 5hmC constitutes between 0.1 and 7% of modified cytosine in non-central nervous system mouse tissues, and 2) little or no change in global 5mC levels [21]. Thus, a significant and rapid loss of 5hmC is observed upon adaptation of MEF cells to culture, which does not translate into a substantial global loss or gain of 5mC. Finally, loss of 5hmC was confirmed at the single cell level by co-staining of cells with 5mC and 5hmC antibodies during adaptation to culture (Figure 1d). In MEF tissue and cultured MEFs (P0, P1 and P2 the 5mC antibody detects bright spots that are coincident with DAPI-positive centromeric heterochromatin (Figure 1d). In contrast, the 5hmC antibody does not stain DAPI-positive centromeric heterochromatin in MEF tissue but instead occupies 5mC-low staining regions that are consistent with 5hmC’s reported euchromatic localization in ES cells [22]. The merged image illustrates 5mC/5hmC counter-staining in MEF tissue (Figure 1d). In agreement with the dot-blot analysis, detection of 5hmC by immunofluorescence is reduced to the extent of being undetectable under the conditions used in P0, P1 and P2 MEFs (Figure 1d and data not shown).

Quantitative RT-PCR (qRT-PCR) of key genes in the DNA methylation cycle revealed a gradual reduction in *Tet* gene expression in culture, with *Tet1* showing a significant decrease in expression compared with primary MEF tissue (Figure 1e). Thus, loss of 5hmC may, in part, be due to loss of Tet enzyme levels in cultured cells. However, it is unlikely that the substantial global loss of 5hmC in culture can be wholly explained in terms of the reductions in *Tet* gene expression levels reported here. DNA replication would be required for passive loss of 5hmC; thus, any increase in proliferation rate in culture in combination with reduced levels of Tet enzymes would accelerate the observed loss of 5hmC. Several recent studies have also reported that addition of vitamin C, a co-factor in Tet-mediated conversion of 5mC to 5hmC, results in increased Tet enzymatic activity and global 5hmC content in mouse ES cells and MEFs [23,24]. Thus, the loss of global 5hmC observed upon adaptation of MEFs to culture may also result from absence of vitamin C, which was not present in the culture media used. Addition of vitamin C to the culture medium during MEF establishment resulted in partial recovery of global 5hmC levels (Figure 1f). This is entirely consistent with the observed reduction of *Tet* expression (Figure 1e); although vitamin C increases Tet enzymatic activity and consequently 5hmC content, insufficient Tet1 enzyme is present in the cultured cells to generate the 5hmC levels observed in the MEF tissue (Figure 1f).

To confirm our observation in an unrelated tissue, naïve CD4^+^ T cells were isolated from two wild-type mouse spleens by magnetic activated cell-sorting (MACS) and cultured under standard stimulating conditions (anti-CD3 and anti-CD28 ± IL-2) for five days. The proliferation of stimulated T cells in culture was confirmed by carboxyfluorescein diacetate succinimidyl ester staining (CFSE, Figure S1a in Additional file 1) and cell cycle staging was assessed by propridium iodide staining (Figure S1b in Additional file 1). In complete agreement with the observations made during adaptation of MEFs to cell culture, a substantial reduction (more than five-fold) in global 5hmC levels was observed in proliferating CD4^+^ T cells after three days of culture (Figure 1g,h).

Loss of 5hmC in a pure CD4^+^ T-cell population suggested that changes in tissue heterogeneity did not contribute to the global loss of 5hmC in culture. To exclude changes in population substructure as a contributing factor to the observed 5hmC loss during MEF establishment, we used both transcriptome profiling and immunohistochemistry. Using published data sets of gene expression in 90 different mouse tissues and cell lines, we identified MEF-specific genes that are a signature of its cellular identity [25] (Gene Expression Omnibus (GEO) ID GSE1133). We identified 20 MEF-specific genes that 1) were most highly expressed in MEFs and 2) whose expression in MEFs was at least two-fold higher than that in any other tissue (Table S1 and Figure S2a in Additional file 1). The majority of the MEF-specific gene set (12/20) showed no reduction in expression between the tissue and cultured MEFs (Figure S2b in Additional file 1), consistent with the tissues having the same cellular identity and origin. The remaining MEF signature genes (8/20) were highly expressed, but to differing extents, in both sets of samples, which may be due to adaptation to culture. This implies that the heterogeneity index of the ‘tissue’ sample is low and it is not highly contaminated with non-MEF cells. In addition, we performed immunostaining of primary embryonic fibroblast preps before and after 1 week in culture with the MEF-specific marker, Arid5b (Figure S2a in Additional file 1). Arid5b staining was equivalent in both tissue and cultured populations (Figure S2c in Additional file 1). Significantly, we observed no obviously negative staining cells in either the tissue or cultured samples, suggesting that selection of a subpopulation of cells in culture is not a contributory factor to the observed global loss of 5hmC. Taken together, these observations, in combination with the overall stability of the 5mC profiles, implies that the primary MEF tissue and cultured MEFs have the same cellular origin but have acquired altered epigenetic signatures.

### Cell culture-induced 5hmC loss occurs genome-wide and independent of 5mC levels

**Figure 2:**
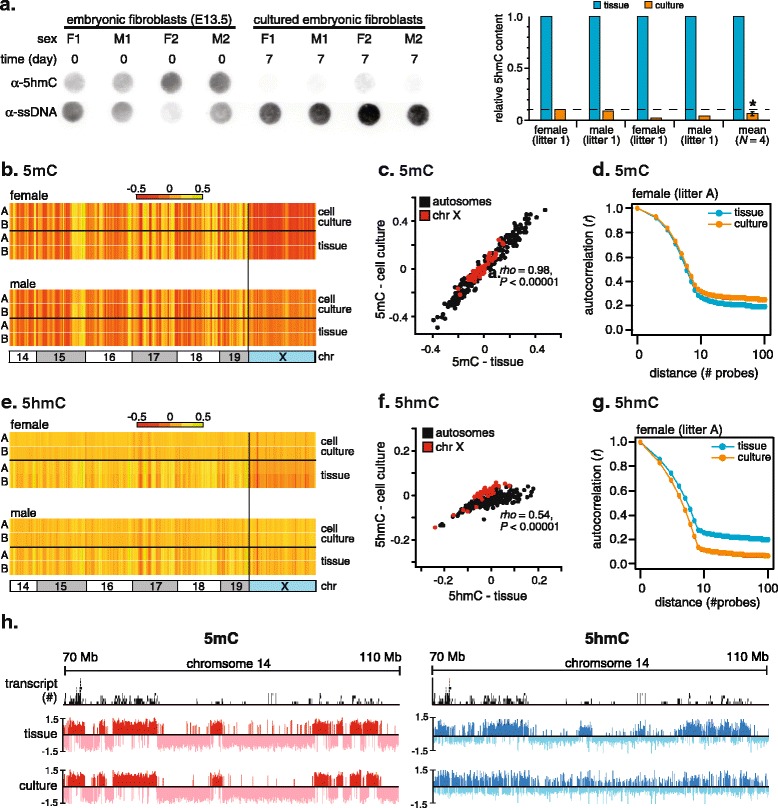
**Cell culture-induced 5hmC loss occurs genome-wide and does not reflect changes in 5mC. (a)** Dot-blot of genomic DNA (500 ng) from MEF primary tissue and cultured cells derived therefrom with a 5hmC-specific antibody (left). Quantification of global 5hmC levels by densitometric analysis of dot-blots (right). 5hmC values were normalized to the single-stranded DNA (ssDNA) loading control. *P < 0.05, Mann-Whitney U-test. **(b)** Heatmap showing median 5mC enrichment levels in non-overlapping 2 Mb windows of the mouse genome (chromosomes 14 to 19 and X) from primary MEF tissue and cultured cells derived therefrom, as determined by MeDIP-chip. **(c)** 5mC-enrichment was highly correlated between MEF tissue and matched cultured cells derived therefrom (Spearman’s *rho* = 0.98, *P* < 0.00001). A scatterplot of 5mC enrichment in male replicate ‘A’ before and after cell culture. **(d)** Autocorrelation analysis of 5mC enrichment of all probes (2.1 million) on the array. **(e)** Heatmap showing median 5hmC enrichment levels in non-overlapping 2 Mb windows of the mouse genome (chromosomes 14 to 19 and X) from primary MEF tissue and cultured cells derived therefrom, as determined by hMeDIP-chip. **(f)** A scatterplot of 5hmC enrichment in male replicate ‘A’ before and after cell culture. **(g)** Autocorrelation analysis of 5hmC enrichment of all probes (2.1 million) on the array revealed a marked reduction in concordance between the 5hmC values of adjacent probes for MEF tissue and matched cultured cells derived therefrom. **(h)** Clear patterning of 5hmC is lost upon adaptation of MEFs to culture. A schematic representation of the 5mC (left panel, red) and 5hmC (right panel, blue) array profiles over a 40 Mb section of chromosome 14; adapted from the UCSC Genome Browser. Refseq transcripts (transcript) mapped to the region are also shown.

The conspicuous loss of global 5hmC observed between primary tissues and their matched cultured cells could reflect genome-wide loss of 5hmC or loss from distinct genomic compartments (that is, repetitive elements, gene-bodies or promoters,) or chromosomes (that is, the inactive X chromosome). To address this, we used a combination of immunoprecipitation and genome-wide tiling microarrays (hMe- and Me-DIP-chip) to determine both the 5hmC and 5mC profiles of four male and female (*N*_male_ = 2, *N*_female_ =2) primary E13.5 MEF tissues and cultured cells derived from the same four samples (*N*_male_ = 2, *N*_female_ = 2) after 7 days in culture (Figure 2). MEF establishment was chosen as the model system to study genome-wide changes in 5hmC patterns as MEFs possess substantially higher levels of 5hmC compared with naïve CD4^+^ T cells (Figure S1c in Additional file 1), enabling more sensitive detection of changes in 5mC and 5hmC by Me- and hMe-DIP assays. An early passage (Figure 2a) was assayed in order to identify early epigenetic changes, and to minimize the effects of prolonged culture and replicative senescence. All male and female replicate MEFs showed marked approximately 10-fold (Mann-Whitney U-test, *P* = 0.028) loss of 5hmC after 7 days in culture as determined by dot-blot (Figure 2a, right panel). As both genic and non-genic regions of the mouse genome have been shown to possess 5hmC, we used tiling microarrays covering all of chromosomes 14 to 19, and both sex chromosomes (X and Y). The features represented on each array are listed in Table S2 in Additional file 1.

The sensitivity of our experimental approach was demonstrated by the ability to detect locus-specific, allele-specific increases in DNA methylation at gene promoters in female cells previously reported to undergo promoter hypermethylation on the inactive X chromosome (X*i*) as part of the dosage compensation mechanism (Figure S3a in Additional file 1). Conversely, the promoter of the *Xist* gene, which is expressed and unmethylated only on the Xi, showed the expected increase of 5mC in male (X*a*) versus female cells (X*a*, X*i*) (Figure S3b in Additional file 1). The X chromosome profile in female cells had significantly reduced levels of both 5mC and 5hmC compared with the male X chromosome profile (Figure S3c in Additional file 1). As male cells possess a single active X chromosome (*Xa*), the reduced 5mC levels observed across the female X chromosome profile suggest genome-wide hypomethylation on the X*i*, consistent with several previous reports [26-29].

To gain an overview of genome-wide modification changes upon adaptation to culture, chromosomes were divided into 2 Mb windows and the median modification content of each region determined. Both the pattern and levels of genome-wide enrichment of 5mC are maintained upon adaptation of MEFs to culture (Figures 2b,c). Autocorrelation analysis of all probes on the array revealed that concordance between neighboring 5mC probes was similar in both tissue and culture samples, consistent with unchanged levels of 5mC, and thus signal-to-noise ratios, in both conditions (Figure 2d). Thus, the genome-wide profile of 5mC in primary MEFs after 7 days in culture is largely unaltered from that of the tissue of origin. The genome-wide conservation of 5mC patterns between primary MEF tissues and their cultured counterparts also suggests that no major changes in cell composition have occurred in the cultured cells.

In complete contrast to the 5mC profile, the presence of 5hmC was significantly reduced and the corresponding profile dramatically altered in MEFs in culture compared with primary cells; the clear patterning of 5hmC observed for MEF tissue is lost in the matched cultured samples for both male and female cells (Figures 2e,f). Autocorrelation analysis confirmed the reduction in concordance between neighboring 5hmC probes in culture (Figure 2g). The higher noise-to-signal ratio in 5hmC profiles of cultured samples was consistent with a global reduction of 5hmC in those samples. As expected, unsupervised hierarchical clustering grouped the 5mC samples by sex, reflecting 5mC differences on the X chromosome between samples, whereas 5hmC samples clustered by sample history (tissue versus culture; Figure S4 in Additional file 1), reflecting the substantial change in the 5hmC profile upon of adaptation of MEFs to culture. A representative portion of chromosome 14 illustrates the striking change in 5hmC profile induced by culture (Figure 2h). In tissue, both 5mC and 5hmC show similar distributions and are enriched in gene-rich regions. The 5mC profile is largely unchanged before and after culture, with domains of enrichment and depletion clearly conserved in both conditions (Figure 2h, left panel). In contrast, the 5hmC profile is substantially altered in culture (Figure 2h, right panel), with regions of enrichment and depletion less discernible against a higher level of background noise, consistent with substantial loss of 5hmC in culture reported here. This change in 5hmC profile was highly similar in all four biological replicates (Figure S5 in Additional file 1).

We next identified peaks of 5hmC enrichment, defined as any five consecutive probes in which a minimum of four probes had an enrichment score above the 90th percentile, yielding a false discovery rate <0.01. Probes within peaks are henceforth referred to as ‘peak probes’. Significantly less 5hmC peak probes were detected in DNA from cultured samples (Mann-Whitney U-test, *P* < 0.01), whereas the number of 5mC peak probes did not change significantly between tissue DNA and DNA from cultured cells (Figure S6a in Additional file 1). The genomic distribution of 5hmC peak probes revealed enrichment of 5hmC peaks in genic regions, as previously widely reported (Figure S6b in Additional file 1). Although broadly similar, the genomic distribution of 5hmC in culture showed a relative reduction in peak probes from genic regions, in particular, exons and introns (Figure S6b in Additional file 1). Interestingly, the number of 5mC peak probes in gene promoters showed a small but significant increase in culture, possibly reflecting discrete promoter hypermethylation events.

### Genome-wide loss of 5hmC and locus-specific gains in 5mC are consistent with loss of methylcytosine dioxygenase activity in culture

**Figure 3:**
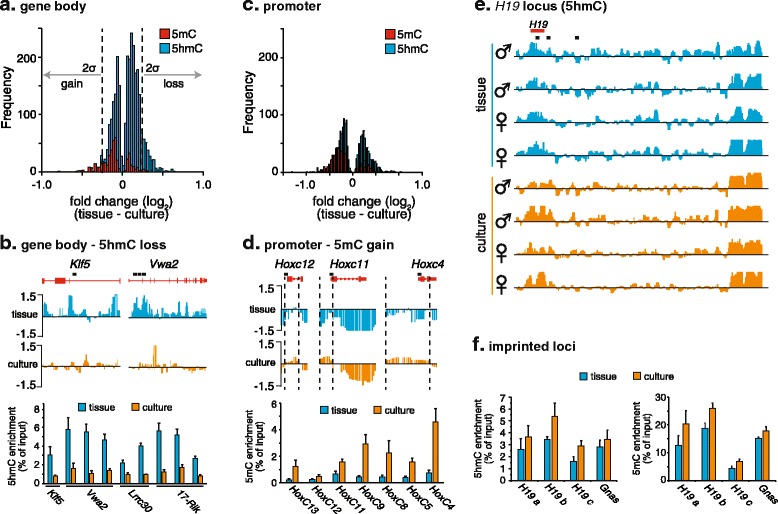
**Global loss of 5hmC and locus-specific gains in 5mC suggest loss of demethylase activity in culture. (a)** Histogram of genes showing a significant change (Mann-Whitney U-test, *P* <0.01) in gene body 5mC and 5hmC content upon adaptation of primary MEF cells to culture. **(b)** Schematic representation of the mean (*N* = 4) 5hmC content of two gene (*Klf5* and *Vwa2*) bodies before and after adaptation to culture. Bar chart of hMeDIP-qPCR showing decreased gene body 5hmC content in cultured MEFs (lower panel). Values are the average of four biological replicates ± standard deviation. Multiple primer pairs were used to determine values across gene bodies of *Klf5*, *Vwa2*, *LRRC30* and *1700054A03Rik* (black bars). **(c)** A histogram of the distribution of genes showing a significant change (Mann-Whitney U-test, *P* <0.01) in promoter 5mC (red) and 5hmC (blue) content upon adaptation of MEF cells to culture. **(d)** A schematic representation of the mean (*N* = 4) promoter 5mC profiles at the *Hoxc-12*, *-11* and -*4* genes before (blue) and after (orange) adaptation to culture (upper panel). Bar chart of hMeDIP-qPCR showing increased promoter 5mC content at the *Hoxc-12*, *-11* and -*4* gene regions in cultured MEFs (lower panel). Values are the average of four biological replicates ± standard deviation. The location of hMeDIP-qPCR primers for each *Hoxc* region is shown in black above gene maps (top). **(e)** The imprinted locus, *H19*, maintains high levels of 5hmC in culture. A schematic representation is shown for the 5hmC profile across the imprinted locus, *H19*, for all eight samples (four male (♂) and four female (♀)). The location of hMeDIP-qPCR primers is shown in black. **(f)** Bar-chart of hMeDIP-qPCR showing retention of 5mC and 5hmC at the imprinted loci *H19* and *Gnas* (lower panel) in cultured MEFs. Values are the average of four biological replicates ± standard deviation.

In contrast to 5mC, 5hmC is enriched in euchromatin, and increased genic 5hmC is broadly associated with increased transcription levels [6,16,22]. Thus, we next sought to (a) examine changes in 5hmC at the level of individual genes and (b) determine the effect of this change on associated transcript levels. The average 5hmC levels of 2,727 gene bodies (42% of all genes analyzed) were significantly altered (Mann-Whitney U-test, *P* < 0.01, adjusted for multiple testing) in culture in comparison to 459 gene bodies (7% of all genes analyzed) showing significantly changed 5mC levels (Figure 3a). Interestingly, the direction of change of genic 5hmC and 5mC levels were contrasting. As expected, 81% of those genes showing both significant (Mann-Whitney U-test, *P* < 0.01) and substantial (top 5% absolute change) changes in genic 5hmC levels in culture also showed loss of 5hmC. Genes losing 5hmC in culture were not enriched for any functional categories (*P* < 0.05) as determined by gene annotation enrichment analysis (data not shown), consistent with a general loss of methylcytosine dioxygenase activity in culture. We confirmed the loss of 5hmC across the gene body of several of the top changing genes, including von Willebrand factor A domain containing 2 (*Vwa2*), Kruppel-like factor 2 (*Klf5*), leucine rich repeat containing 30 (*Lrrc30*) and a non-coding RNA (1700054A03Rik) by hMeDIP-qPCR in all eight biological replicates (Figure 3b). The observed loss of 5hmC from individual genes was highly consistent between replicates (Figure S7 in Additional file 1). In contrast, 85% of those genes showing both significant (Mann-Whitney U-test, *P* < 0.01) and substantial (top 5% absolute fold change) changes in genic 5mC levels in culture showed gain of 5mC in culture (Figure 3a). Loss of a demethylase activity has been previously proposed as the cause of promoter hypermethylation events commonly observed in transformed cell lines and cancer cells, and inhibition of Tet methylcytosine dioxygenase activity is directly linked with increased 5mC, while over-expression of Tet1 leads to global demethylation [11,30]. Focusing on promoter regions (representing 0.6% of the array), we observed a shift towards gain of 5mC but not 5hmC at gene promoters in culture (Figure 3c). Although no enrichment for individual Gene Ontology (GO) terms was observed for promoters gaining 5mC in culture after correction for multiple testing, functional annotation clustering of the GO terms revealed an enrichment for genes involved in ‘pattern specification’ and ‘organ development’ (Table S4 in Additional file 1). The genes underlying these terms were primarily homeobox genes, including *Hoxc4*, *Hoxc6*, *Nkx2-3*, *Cdx1* and *Rax*. Indeed, an agglomerative methylation event was evident across the entire *HoxC* cluster upon adaptation to culture, with most *HoxC* genes showing a gain in promoter methylation (Figure 3d; Figure S8 in Additional file 1). Comparative bisulfite sequencing analysis of the *Hoxc4*, *Hoxc5*, *Hoxc9*, *Hoxc11* and *Hoxc12* promoters in the tissue and cultured MEF samples is consistent with gains of 5mC at all promoters tested, with a median gain of 18% (range: 2.1 to 26%; Figure S8b in Additional file 1). It is noteworthy that both the global loss of 5hmC and the gain of promoter-specific hypermethylation observed here are hallmarks of many cancers, and loss of a TET-mediated demethylase activity through mutation or substrate inhibition is being implicated in a growing number of malignancies [31]. Thus, adaptation of mammalian cells to culture may offer a tractable model system in which to study the initiation and progression of epigenetic dysfunction upon loss of a methylcytosine dioxygenase activity.

It has been reported that 5hmC DNA immunoprecipitation can be compromised by the effects of DNA sequence composition on antibody affinity [32,33]. To confirm that the observed changes in 5hmC enrichment were independent of the enrichment technique, we assayed loci that increased, decreased or retained 5hmC in culture using GLIB (glucosylation, periodate oxidation, biotinylation) enrichment followed by quantitative PCR (qPCR) [34]. The relative enrichment of 5hmC at all tested loci was almost identical when using either GLIB or hMeDIP enrichment (Figure S9a,b in Additional file 1). Moreover, the genome-wide profiles of 5hmC determined here were highly similar to a recently published whole-genome 5hmC profile of primary MEFs determined by GLIB-seq (Figure S9c in Additional file 1; Short Read Archive accession SRX244234) [35].

Intriguingly, against a background of global loss of 5hmC, the imprinted loci, *H19* and *Gnas*, remained highly enriched for 5hmC in culture (Figure 3e,f). This observation mirrors that seen in the developing embryo where *H19* and other imprinted loci are protected from demethylation, suggesting that 5hmC dynamics at such loci differ from those in the remainder of the genome [36]. Similar enrichment patterns were observed across the *H19* and *Gnas* loci in a recently published study of 5hmC patterns in cultured MEFs using GLIB-seq (Figure S9d in Additional file 1) [35]. In agreement with this observation, during vitamin C induced, Tet-dependent demethylation of promoters in mouse ES cells, many imprinted genes, including *H19* and *Gnas*, remain resistant to demethylation [23].

Whereas the tiling microarrays used here assay 25% of the mouse genome, they do not represent a complete genome-wide survey of 5hmC changes in MEFs during adaptation to culture. To confirm the observed redistribution of 5hmC on a genome-wide scale, we subjected both input and hMeDIP DNA isolated from MEFs before and after adaptation to culture to massively parallel sequencing (hMeDIP-Seq). A minimum of 30 million uniquely mapped reads were obtained for each sample (Figure S10a in Additional file 1). The results obtained from this additional biological replicate of MEF establishment in culture were highly similar to those determined by array hMeDIP-chip (*r* = 0.4, *P* < 1 × 10^-16^; Figure S10b in Additional file 1), including loss of 5hmC from gene bodies (Figure S10c in Additional file 1), maintenance of 5hmC across the *H19* locus (Figure S10d in Additional file 1) and widespread gains in 5hmC across the *Hoxc* cluster (Figure S10e in Additional file 1). Interestingly, gains in 5hmC were observed across all *Hox* clusters, suggesting that these loci are particularly susceptible to epigenetic disruption in culture (Figure S10f in Additional file 1). Finally, only 45% of peaks of 5hmC enrichment observed in tissue were also observed in the cultured samples, again confirming the genome-wide redistribution of 5hmC in cultured cells.

### Cell culture results in repression of epigenetic processes

**Figure 4:**
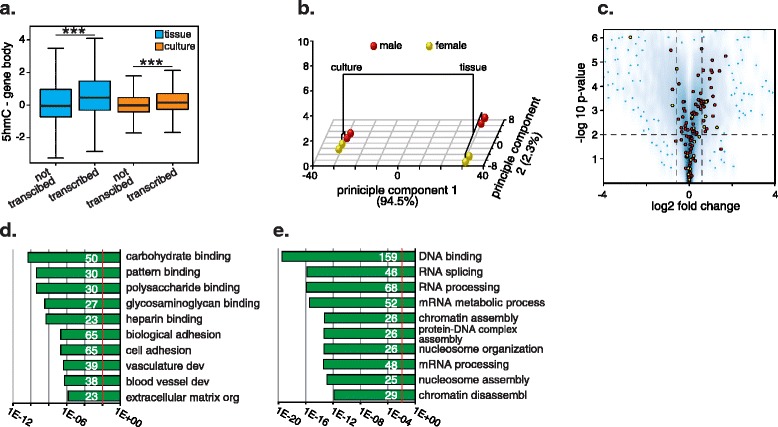
**Change in cell state accompanies adaptation of cells to culture. (a)** A boxplot showing that 5hmC is enriched in transcribed genes in MEF tissue (blue) and cultured (orange) cells. A gene was classified as expressed if its probes had a combined detection *P*-value <0.01. ***, *P* < 0.01, Mann-Whitney U-test. **(b)** Hierarchical clustering of the principle components of gene expression microarray data upon adaptation of MEF tissue to culture showing that the transcriptome of cultured MEFs is very different from that of the MEF tissue from which they were derived. Tissue samples and culture samples cluster separately. Adaptation to culture also results in a reduction in the sex-specific transcriptional differences between samples. **(c)** Volcano plot showing the widespread, bi-directional changes in gene expression observed upon adaptation of MEF tissue to culture conditions. Red dots indicate genes losing 5hmC in culture; yellow dots indicate genes gaining 5hmC in culture; blue dots indicate the 100 most outlying genes. The horizontal dashed line indicates *P* < 0.01; the vertical dashed lines indicate fold change >1.5. **(d)** Bar chart showing the GO term analysis of genes up-regulated (*P* < 0.01, fold-change >1.5) for MEFs in culture. The number of individual genes in each category is shown in white. Red line, *P* = 0.01. **(e)** Bar chart showing GO term analysis of genes down-regulated (*P* < 0.01, fold-change >1.5) for MEFs in culture were significantly enriched for epigenetic processes. The number of individual genes in each category is shown in white. Red line, *P* = 0.01. The x-axis values for **(d)** and **(e)** are '-log10(P-value)'.

Genic 5hmC levels are generally positively associated with transcriptional activity [6,16]. Thus, we sought to determine if the observed genome-wide loss of 5hmC reflected changes in transcription. RNA from the same samples used for genome-wide 5mC/5hmC profiling (*N* = 8; Figure 2a) were labeled and hybridized to Illumina MouseWG-6 v2.0 gene expression microarrays. Despite the loss of 5hmC in culture, a positive association with transcription level was observed in both cultured cells and MEF tissue (Figure 4a). Principle component analysis of the gene expression data clearly separated uncultured and cultured samples; the first principle component (PCA1) explained approximately 95% of the variation in the data whereas PCA2 explained 2.3% of the variation, which seemed to reflect sample gender (Figure 4b). Thus, adaptation to culture resulted in an altered transcriptional state that was (i) similar between all four cultured samples, but (ii) differed markedly from that of the MEF tissue from which the cultures were derived. The changes in gene expression induced by adaptation to culture were widespread, with 7,287 genes identified as differentially expressed (*P* < 0.01, paired *t*-test, adjusted for multiple testing). The changes in differentially expressed genes were primarily small (90% changed less than two-fold) and bi-directional; 3,865 genes significantly up-regulated and 3,431 genes down-regulated (Figure 4c). However, gene expression changes explained <1% (*r*^2^ = 0.009, *P* = 0.00001) of the variation in genic 5hmC values between MEF tissue and the cultured cells derived therefrom. Indeed, approximately 40% of genes showing a significant change in genic 5hmC level between MEF tissue and culture were not expressed in either sample type as determined by gene expression microarray. These findings make the important point that the observed genome-wide loss of genic 5hmC did not result from a concomitant genome-wide loss of transcription.

Gene annotation enrichment analysis was used to identify the functional categories of genes changing expression upon adaptation to culture. Using strict criteria for definition of differential gene expression (paired *t*-test *P* < 0.01, absolute fold-change >1.5) we found that up-regulated and down-regulated genes were significantly enriched (modified Fisher exact test, *P* < 0.01, corrected for multiple testing) for different functional annotations (Figure 4d,e). Genes up-regulated in culture were enriched for carbohydrate metabolism and cell adhesion genes, possibly reflecting adaptation to growth on a two-dimensional plastic surface (Figure 4d). However, a very recent study found that genes belonging to the categories ‘cell adhesion’ and ‘biological adhesion’ were also the most significantly down-regulated upon over-expression of Tet1 during re-programming of MEFs to induced pluripotent cells, suggesting that these genes are directly regulated by Tet1 [37]. In striking contrast, genes down-regulated in culture were highly significantly enriched (*P* < 1e^-12^) for categories linked with chromatin structure, chromatin re-modeling, and RNA processing (Figure 4e). Most of these functional categories contained overlapping sets of genes, including methylation-sensitive DNA binding proteins (*Ctcf*, *Cxxc1*, *Cxxc4, Gpbp1*), DNA methyltransferases (*Dnmt1* and *Dnmt3b*), chromatin remodeling enzymes (*Smarcad1*), histone-modifying enzymes (*Ezh2*, *Hdac2*), and multiple members of the histone H1, H2a and H2b gene families. Interestingly, the *Tet1* gene was also significantly down-regulated (paired *t*-test, *P* < 0.01, fold-change = -1.4). Gene set enrichment analysis confirmed the significance of PRC2 component (*Ezh1*, *Ezh2*, *Eed*, *Suz12* and *Rbbp4*) down-regulation in culture (*P* = 0.002). Numerous recent studies have revealed extensive crosstalk between DNA methylation and PRC2, with the appropriate deposition of each repressive mark being inter-dependent, linking these two layers of epigenetic regulation [38,39].

### Genome-wide redistribution of 5hmC is a general feature of cell culture

Finally, we sought to determine the generality of the 5hmC redistribution in cultured MEFs by analysis of previously published whole genome profiles of 5mC and 5hmC in mouse CD4^+^ T cells undergoing differentiation in culture (GEO: GSE59212) [40,41]. In a very recent study, Tsagaratou and colleagues [41] used cytosine-5-methylenesulfonate (CMS)-DIP to dissect the dynamics of 5hmC in murine naïve CD4^+^ T cells and T-helper cells derived from those cells *in vitro*. Unlike hMeDIP, the anti-CMS antibody recognizes CMS generated from 5hmC by sodium bisulfite conversion, and exhibits markedly lower background pull-down and less sequence-dependent performance than hMeDIP [42]. First, the authors noted a dramatic and rapid loss of global 5hmC in culture as reported here (Figure 1g). Indeed, the authors noted that the loss of 5hmC in cultured T-helper cells was sufficient to preclude reliable locus-specific analysis with CMS-DIP. On a genome-wide scale, however, the patterns of 5hmC redistribution in CD4^+^ T cells (Figure S11a in Additional file 1) were remarkably similar to those observed in MEFs (Figure 2h). The number of 5hmC-enriched peaks was reduced seven-fold in culture, with a significant (χ^2^; *P* < 0.001) shift of peaks from genic regions to inter-genic regions (Figure S11b in Additional file 1), precisely as seen in MEFs (Figure S6a,b in Additional file 1). Moreover, the median 5hmC content of gene bodies reflected this loss (Figure S11c in Additional file 1). Unlike MEFs, however, CD4^+^ T-cell promoters showed a tendency towards 5hmC loss, though on a smaller scale than that observed for gene bodies (Figure S11d in Additional file 1).

To study genome-wide changes in 5mC in cultured CD4^+^ T cells we analyzed published methyl-binding domain (MBD)-Seq data of mouse CD4^+^ naïve T cells and their cultured derivatives (GEO: GSE25689) [40]. MBD affinity purification interrogates methylated CpG-rich sites across the genome [40]. Whereas gene bodies showed a modest skew towards 5mC loss in culture, promoters were skewed towards gain of 5mC in culture, particularly those promoters undergoing large (greater than two-fold) gains (Figures S11e,f in Additional file 1). Thus, the genome-wide redistribution of 5hmC in cultured CD4^+^ T cells is highly similar to that observed in MEFs, as is the skew towards gains in promoter methylation (5mC). To further validate these findings we performed h/MeDIP-qPCR at loci gaining and losing 5hmC and/or 5mC in MEFs and CD4^+^ T cells. The changes in 5hmC were remarkably similar in CD4^+^ T cells and MEFs, in both the direction and scale of change (Figures S12a,b in Additional file 1). In contrast, 5mC was remarkably stable in both cell types during culture, in agreement with our observations in MEFs (Figures S12c,d in Additional file 1). Finally, we assessed the effect of vitamin C on 5hmC levels during culture of CD4^+^ T cells. The addition of vitamin C resulted in partial or complete rescue of 5hmC at all loci tested (Figures S12b in Additional file 1). Significantly, two loci (*Hoxc4* and *Gnas*) showed two-fold and six-fold increases in 5hmC compared with uncultured, non-proliferating naïve T cells, respectively. Thus, the observed increase in 5hmC induced by vitamin C cannot be due solely to an inhibitory effect on proliferation rate, strongly suggesting increased methyl-cytosine dioxygenase activity.

## Discussion

A fundamental assumption underlying the use of cultured cells in biological research is that cell lines retain and mimic the molecular characteristics of the tissue from which they were derived, allowing observations made *in vitro* to be directly translated to an *in vivo* context. Cell lines have been extensively used to investigate the normal and pathogenic role of DNA methylation, particularly its dysregulation in cancer [20]. However, over 20 years ago, Antequera, Boyes and Bird [11] noted aberrant *de novo* CGI methylation at 14 loci in mouse NIH3T3 and L cells, which was interpreted as cell culture-induced changes in DNA methylation. Subsequently, using restriction landmark genomic scanning, a comparison of six human ES cell lines derived and cultured in various conditions showed widespread inter-line differences in DNA methylation, which could be further altered by changing serum levels in culture [43]. Mouse ES cells are derived from E3.5 blastocyst cells that are globally hypomethylated, but once grown in serum they become hypermethylated [44]. In analogous observation to our adaptation experiment, there is a massive deposition of DNA methylation during ES cell establishment in serum [45]. However, cultivating ES cells in the presence of small molecule inhibitors (2i medium) leads to reversible global hypomethylation [46]. Molecular analysis suggests there is a high degree of similarity between the hypomethylated E3.5 blastocyst cells and 2i ES cells, as well as between hypermethylated E6.5 blastocyst cells and serum ES cells [44,47,48]. This demonstrates that the correct culturing conditions can lead to ‘epigenetic’ equivalence between cells propagated in culture and their embryo/animal counterparts.

Restriction landmark genomic scanning was also used to reveal that CGI hypermethylation was 5-fold to 90-fold more common in cancer cell lines compared with primary malignancies, and that approximately 60% of the loci methylated in cancer cell lines were never methylated in the 114 primary malignancies studied [49]. A recent large-scale genome-wide study of DNA methylation re-emphasized the effect of cell culture on the epigenome [50]. Using reduced-representation bisulfite sequencing, Varley and colleagues [50] found that normal human tissues and primary cell lines form two separate and robust clades based on genome-wide DNA methylation patterns. That is, the methylome of primary cell lines from multiple different tissues more closely resembled each other than the normal tissues from which they were derived [50]. How and why cell lines accumulate CGI methylation in culture is unknown, but in their seminal paper in 1990, Antequera, Boyes and Bird proposed that the observed CGI hypermethylation was consistent with a loss of DNA demethylase activity in culture, 20 years prior to the characterization of a robust mammalian demethylation pathway based on 5hmC/Tet [11].

By studying the establishment of MEFs in culture we found that adaptation by mammalian cells resulted in a rapid and comprehensive re-programming of the transcriptome and epigenome, indicative of an altered cell state. Re-programming involved almost complete loss of 5hmC in cultured cells, consistent with loss of a methylcytosine dioxygenase activity in culture; in the absence of Tet enzymatic activity, 5hmC lost by dilution during each round of DNA replication cannot be restored. This loss was observed genome-wide and was not restricted to particular genomic compartments, again consistent with a general loss of Tet-mediated demethylase activity. In the absence of a demethylase activity, *de novo* methylation events cannot be erased and would result in progressive accumulation of methylation at methylation-prone loci, which over time is likely (perhaps through spreading) to affect gene expression, imprinting, chromatin structure and genome stability. Indeed, even at the early time-points assayed here, we observed increased CGI methylation at several loci known to be prone to hypermethylation in cancer, such as the *Hox* clusters [51,52].

The epigenetic remodeling we observed was paralleled by extensive gene expression changes in culture. Up-regulated genes were linked to cell adhesion and extracellular matrix organization, possibly reflecting adaptation to growth on a two-dimensional plastic surface. However, a recent study has suggested that these genes are regulated directly by Tet1 in MEFs [37]. A highly significant down-regulation of genes involved in a variety of epigenetic processes, including nucleosome assembly, chromatin modification, DNA methylation and DNA demethylation, were also observed. These included *Tet1* and three components of the PRC2 complex, which deposits the repressive histone modification H3K27me3, namely *Ezh2*, *Suz12* and *Rbbp4*. Tet1 directly interacts with Suz12 in mouse ES cells, and loss of Suz12 results in a global loss of 5hmC in mouse ES cells similar in scale to that reported here [35]. It is tempting to speculate that CGIs marked by H3K27me3 are actively maintained in an unmethylated state by Tet1 and may thus be particularly susceptible to aberrant methylation upon loss of the Tet1 interaction with the PRC2 complex, or methylcytosine dioxygenase activity in culture.

A 50% reduction in *Tet1* gene expression is unlikely to fully account for the significant global loss of 5hmC observed, particularly as expression of *Tet2* and *Tet3* were relatively unchanged in culture. All Tet enzymes require 2-oxoglutarate and vitamin C as cofactors to efficiently catalyze hydroxylation of canonical 5mC to 5hmC [24]. Most cell culture media do not contain vitamin C, and several recent studies have shown that addition of vitamin C increases Tet enzyme activity and 5hmC levels in MEFs and mouse ES cells [23,37]. We observed a partial yet substantial recovery of global 5hmC levels upon addition of 1,000 μM vitamin C during establishment of MEF cultures. This result suggests that the observed loss of 5hmC results from both a reduction of *Tet1* levels and general loss of Tet activity due to limiting co-factors. Significantly, addition of vitamin C to mouse ES cells resulted in greater Tet-dependent demethylation at methylated CGIs, resulting in a more blastocyst-like state in the ES cells [23]. This finding, in combination with our observations reported here, suggest that optimization of culture media with regard to enzymatic co-factors may ameliorate the scale of epigenetic changes occurring in culture. The identification of suitable substrates for culturing cells, probably in three dimensions, will also lead to improvements in generating *in vitro* equivalents of *in vivo* cellular morphologies. As vitamin C is an essential co-factor for a growing list of enzymes, including the epigenetic modifiers JHDM1B, KDM3 and KDM4, and regulates hypoxic inducible factor (HIF)-1 activity through asparagine and proline hydroxylases and is also critical to collagen metabolism, caution may need to be applied when assessing the effect of vitamin C on individual molecular pathways such as DNA demethylation [53-55].

Our findings reinforce a growing realization that cell line models of human diseases, particularly cancer, can be poor surrogates for many aspects of *in vivo* biology, as emphasized in a recent study of multi-drug resistance genes in ovarian cancer, in which no correlation in expression was found between primary ovarian cancer samples and established cancer cell lines [56]. Though novel, the findings presented here do not address cause and effect of the observed reprogramming during the process of adaptation of cells to culture. That is, are the observed changes driven by epigenome dynamics or is the epigenome responding to changes in transcription factor networks? Are the observed gains in 5mC due to loss of 5hmC or independent of this deficit? A system in which Tet1 expression can be induced during the process of adaptation to culture would help to address some of these questions. It is worth noting that Tet1 can replace Oct4 to initiate somatic cell reprogramming in conjunction with Sox2, Klf4, and c-Myc [57]. This requires an active form of the enzyme that results in altered DNA methylation and hydroxymethylation profiles in the derived pluripotent cells.

## Conclusion

Our study demonstrates the utility of using 5hmC profiling as a read-out of cellular state [31]. It is possible that this type of analysis can be applied to many dynamic systems, including models for cancer progression [58]. In conclusion, over 20 years since its initial proposal [11], we present the first clear evidence that cell culture-induced hypermethylation results from loss of a demethylase activity, and that the changes induced by cell culture are far more rapid and widespread than previously thought. It will be of future interest to determine if these changes are reversible in novel culturing media [59].

## Materials and methods

### Establishment of mouse embryonic fibroblast cultures

MEF cultures were established from wild-type B6 13.5 days-post-coitum embryos. The excised uterus containing the embryos was transferred to a 25 ml universal tube containing cold phosphate-buffered saline (PBS)/PS solution (1× PBS and 5% penicillin/streptomycin). The embryo string was transferred to a 10 cm petri dish containing 5 ml PBS/PS solution and the placenta, membranes, string and organs were discarded. The head was retained for embryo sexing by PCR analysis (see below). The resulting material was finely chopped with a scalpel blade and incubated in 300 μl trypsin per embryo for 10 minutes at 37°C. The tissue was further homogenized by repeated (20 times) syringing (19G needle and 3 ml syringe). After the addition of 5 ml growth medium (Dulbecco’s modified Eagle medium, Invitrogen, Paisley, Renfrewshire, UK, catalogue number 41965-039 15% fetal calf serum (FCS); 5% penicillin/streptomycin; 5% sodium pyruvate solution, Invitrogen catalogue number S8636; 5% MEM non-essential amino acid solution, Invitrogen catalogue number M7145), cells were briefly centrifuged (300 × g for 4 minutes) and re-suspended in 5 ml growth media and plated onto a T25 cell culture flask.

Embryos were sexed by PCR as previously published [60]. Briefly, embryo heads were placed in a 1.5 ml eppendorf tube with 500 μl H_2_O and boiled for 10 minutes, vortexed vigorously and spun for 1 minute at 10,000 × g in a bench top centrifuge. The resulting supernatant (2.5 μl) was then used as the template in the PCR amplification of a fragment of the *Ube1X* (chromosome X) and *Ube1Y* (chromosome Y) genes, using the following primers (5′-3′): TGGTCTGGACCCAAACGCTGTCCACA and GGCAGCAGCCATCACATAATCCAGATG. PCR products were run on a 2.5% agarose gel.

### Naïve CD4^+^ T cell isolation and culture

Naïve CD4^+^ T cells were isolated from B10.PLxC57BL/6 mice by MACS according to the manufacturer’s instructions (Miltenyi Biotec, Bergisch Gladbach, North Rhine-Westphalia, Germany and cells were cultured at a density of 3 × 10^6^/ml on six-well plates for five days in RPMI 1640 medium supplemented with 2 mM L-glutamine, 100 U/ml penicillin, 100 μg/ml streptomycin, 5 × 10^-5^ M 2-ME (all from Invitrogen Life Technologies, Paisley, UK), and 10% FCS (Sigma, Irvine, Scotland For T-cell stimulation, cells were cultured at a density of 1.5 × 10^6^/ml in the presence or absence of 10 U/ml rIL-2 (R&D Systems, Abingdon, Oxfordshire, UK on six-well plates pre-coated with 2 μg/ml anti-CD3 (clone 145.2C11; eBioscience, Hatfield, Hertfordshire, UK) plus anti-CD28 (clone 37.51; e-Bioscience).

### Cell division analysis: CSFE

Isolated CD4^+^ T cells were labeled with 5 μM carboxyfluorescein diacetate succinimidyl ester (CFSE; Sigma) at 37°C for 20 minutes. Cells were washed in RPMI 1640 supplemented with 10% FCS and cultured as stated above. At the indicated time-points, cells were harvested and stained with anti-CD4-eFluor450 (clone RM4-5; eBioscience). Flow cytometric data were acquired using a BD LSRFortessa cell analyzer (Becton Dickinson, Oxford, Oxfordshire, UK and data analyzed using FlowJo software (Treestar version 3.2.1, Ashland, Oregon, USA).

### Cell cycle analysis: propidium iodide

Cells were sampled at the indicated time-points and single-cell suspensions were immediately fixed in 2% paraformaldehyde for 20 minutes at 37°C prior to surface staining with anti-CD4-eFluor450. Cells were then re-suspended in ice-cold 90% methanol and stored at -20°C. Cells were washed extensively in PBS containing 2% FCS (Sigma) and stained with propidium iodide (eBioscience) for 30 minutes at 4°C. Flow cytometric data were acquired using a BD LSRFortessa cell analyzer (Becton Dickinson) and data analyzed using FlowJo software (Treestar version 3.2.1).

### DNA immuno-dot-blot

DNA samples were added to denaturation buffer (0.4 mM NaOH, 10 mM EDTA) and denatured for 10 minutes at 100°C. Samples were rapidly chilled for 5 minutes on wet ice and then applied to a positively charged nylon membrane under vacuum using a 96-well Dot Blot Hybridisation Manifold (Harvard Apparatus Limited, Holliston, MA, USA The membrane was washed twice in 2× SSC buffer, UV-crosslinked, and dried for 1 h at 70°C. Duplicate membranes were probed with antibodies specific to 5mC (Eurogentec, Seraing, Liege, Belgium; dilution factor 1:2,000) and 5hmC (Active Motif, Carlsbad, CA, USA dilution factor 1:8,000). To control for loading, duplicate membranes were either probed with a rabbit poly-clonal antibody raised against single-stranded DNA (Demeditec Diagnostics, Kiel, Schleswig-Holstein, Germany) or stained with methylene blue. Subsequently, membranes were probed with either a rabbit (α-5hmC and α-ssDNA membranes) or mouse (α-5mC membranes) IgG antibody conjugated to horseradish peroxidase. Following treatment with enhanced chemiluminescence substrate, membranes were scanned on an ImageQuant LAS 4000 (GE Healthcare, Little Chalfont, Buckinghamshire, UK) imaging station. Spot intensity was quantified using ImageJ image processing and analysis software (NIH).

### 5-Methylcytosine and 5-hydroxymethylcytosinse immunodetection in fibroblasts

Immunofluorescence staining of MEFS was carried out as previously described [9,33] with 5mC mouse monoclonal (Eurogenentec) and 5hmC polyclonal (Active Motif) antibodies.

### Arid5b immunodetection in fibroblasts

Embryonic fibroblast cells or primary embryonic fibroblasts (CD1 and C57BL/6) on glass coverslips coated with gelatin were stained using standard immunocytochemistry protocols. Cells were fixed with 4% paraformaldehyde for 10 minutes at room temperature and incubated with anti Arid5b antibody (Novus Biologicals, Littleton, Colorado, USA) followed by a 1 hour incubation with Alexafluor 488 secondary antibody (Life Technologies, Life Technologies, Paisley, Renfrewshire, UK prior to staining with DAPI (4′,6-diamidino-2-phenylindole). Imaging was done using a Zeiss Axioskop 2 microscope and Zeiss objectives.

### Immunoprecipitation and genomic mapping of 5mC- and 5hmC-containing DNA

Genomic DNA (5hmC-IP; 2.5 μg in 450 μl of TE), sonicated to yield a fragment distribution of 300 to 1,000 bp, was denatured by incubation for 10 minutes at 100°C. Samples were rapidly chilled on wet ice. At this point, 45 μl (10%) of denatured sample was removed and saved as input, and 45 μl of 10× immunoprecipitation (IP buffer (100 mM Na-phosphate at pH 7.0 (mono and dibasic), 1.4 M NaCl, 0.5% Triton X-100) and 1 μg of α-5hmC (Active Motif, catalog number 39769) antibody were added to the remaining sample. Samples were incubated overnight at 4°C with gentle agitation. Then, 40 μl of magnetic beads (Dynabeads Protein G; Invitrogen) in 1× IP buffer was added to each sample to allow magnetic separation of the antibody from the unbound DNA using a magnetic tube rack. Samples were incubated for 1 h at 4°C with gentle agitation. Beads were collected with a magnetic rack and washed with 1 ml of 1× IP buffer for 10 minutes at room temperature with gentle agitation; washing was repeated three times. Beads were collected with a magnetic rack and resuspended in 250 μl of digestion buffer (50 mM Tris at pH 8.0, 10 mM EDTA, 0.5% SDS) followed by addition of 10 μl of proteinase K (20 mg/ml; Roche Applied Science, Penzberg, Upper Bavaria, Germany) and incubation overnight at 52°C with constant shaking (800 rpm). Finally, beads were removed using a magnetic rack, and DNA was purified from the remaining sample using a QIAquick PCR Purification Kit (QIAGEN, Hilden, North Rhine-Westphalia, Germany eluting in a final volume of 47 μl of dH_2_O. Inputs were also purified using a QIAquick PCR Purification Kit and eluted in 47 μl of dH_2_O. Subsequently, 10 ng of input and IP DNA was subjected to whole genome amplification using the GenomePlex Complete Whole Genome Amplification Kit (Sigma-Aldrich, St. Louis, Missouri, USA as per the manufacturer’s instructions. Amplified DNA was run on a 1.2% agarose gel to confirm consistency of fragment size between samples. Subsequently, amplified DNA samples were Cy5 (IP) or Cy3 (input) labeled by random priming using the Dual-Color DNA Labeling Kit (NimbleGen (Hoffmann-La Roche), Basel, Basel-Stadt, Switzerland Labeled samples were applied to NimbleGen mouse (mm8) whole genome tiling array (covering chromosomes 14 to Y) and hybridized overnight at 42°C. Slides were washed and scanned as per the NimbleGen protocol.

### Analysis of microarray data

All analysis of NimbleGen microarray data was performed using custom-written scripts implemented in the statistical programming language R. First, each array was subjected to locally weighted scatterplot smoothing (Loess normalization)). The data were then smoothed using a running median in a sliding window of seven probes (approximately 1,700 bp). A peak of 5hmC was defined as any region of five consecutive probes in which at least four probes had an enrichment value greater than the 90th percentile. This definition of a peak resulted in a false discovery rate of <0.01. Hierarchical clustering was performed using the ‘hclust’ function in the R programming language; a distance matrix was computed for each data set using Euclidean distance; subsequently samples were clustered using Ward’s minimum variance agglomeration method. Autocorrelation analysis was carried out using the ‘acf’ function of the R stats package. All R scripts are available upon request (to CEN).

### hMeDIP-Seq

hmeDIP-Seq was performed as previously described [61]. Briefly, DNA was sonicated to yield fragmented DNA with a modal size of 150 bp. Following end-repair and A-tailing, Illumina TruSeq adaptors were added to the resulting DNA according to the manufacturer’s instructions. The adaptor ligated DNA was then subjected to hMeDIP as outlined above. The resulting input and hMeDIP samples were PCR amplified for 15 cycles using single-end Illumina PCR primers. Finally, libraries were size selected by agarose gel electrophoresis. All DNA purification steps were carried out using Agencourt AMPure Beads (Beckman Coulter, Brea, California, USA), and DNA quality, quantity and size were tested after every step on an Agilent Bioanalyser using High Sensitivity DNA chips (Agilent Technologies, Santa Clara, California, USA Samples were subjected to single-end sequencing on the Illumina GAIIx platform. Sequencing was carried out at the Genomics Core, Albert Einstein College of Medicine, New York.

### Analysis of hMeDIP-Seq data

hMeDIP-seq reads were generated on an Illumina Genome Analyser IIx and mapped to the mm9 build of the mouse genome using the bowtie short read aligner [8]. Where a read mapped to a number of potential locations, only the best hit was retained (in terms of the number of mismatches and their qualities; as specified by the bowtie ‘-best’ parameter).

BEDTools [9] was used to count mapped reads that overlap genomic windows, calculate coverage, and normalize data to total read count. The MACS program was used for peak calling (default parameters).

### Analysis of CMS-Seq data

Normalized (reads per million) CMS-Seq data for mouse naïve CD4^+^ T cells and their *in vitro* derived T-helper type 1 (Th1) counterparts were downloaded from the GEO (accession GSE59212) [41]. Peaks of 5hmC (CMS) enrichment were determined using MACS ChIP-Seq peak-calling software with default parameters. Gene-body and promoter enrichment was defined as median coverage across the entire genic region. The BEDtools suite of software was used to map enriched peaks to different genic compartments based on mouse genome version 9 (mm9) refGene coordinates [62]. Genome track images were modified from the Integrated Genome Viewer software [63].

### Analysis of MBD-Seq data

Normalized (reads per million) MBD-Seq data for mouse naïve CD4^+^ T cells and their *in vitro* derived T-helper type 1 (Th1) counterparts were downloaded from the GEO (accession GSE25689) [40]. Peaks of methyl-CpG enrichment were determined using MACS ChIP-Seq peak-calling software with default parameters. Gene-body and promoter enrichment was defined as median coverage across the entire genic region. Promoters and genes with less than five reads in both samples were excluded. The BEDtools suite of software was used to map enriched peaks to different genic compartments based on mouse genome version 9 (mm9) refGene coordinates [62]. Genome track images were modified from the Integrated Genome Viewer software [63].

### GLIB enrichment of 5hmC

GLIB enrichment was performed using the Hydroxymethyl Collector Kit (Active Motif). Genomic DNA was sonicated (Bioruptor; Diagenode, Liege, Seraing, Belgium to produce DNA fragments ranging in size from 200 to 1,000 bp, with a modal fragment size of 400 bp. Fragmented DNA (1 μg) was glucosylated by incubation with dUTP containing a modified azide glucose group and 20U β-glucosyltransferase for 1 h at 37°C. The modified glucose group was then biotinylated and fragments of DNA containing modified biotin-azide-glucose-5hmC were purified with magnetic streptavidin beads. DNA was then passed through a DNA purification column and eluted in elution buffer prior to qPCR. All reagents employed were supplied with the Hydroxymethyl Collector Kit (Active Motif).

### DNA and RNA extraction and quantitative reverse transcriptase PCR

DNA was isolated from cells and tissues using the QIAGEN DNeasy Mini Kit. Genomic DNA was isolated from mammalian cells using the following method. The cell pellet was resuspended in 200 μl lysis buffer (20 mM Tris pH8, 4 mM EDTA, 20 mM NaCl, 1% SDS). Proteinase K (20 μl, 20 mg/ml; Roche Applied Science) was added and samples incubated overnight at 55°C. The second day samples were cooled down to 37°C and treated with 1 μl RNase cocktail (Ambion; AM2286) and incubated at 37°C for 30 minutes. Genomic DNA was isolated by standard phenol/chloroform extraction. RNA was extracted from cell lines using TRIzol reagent (Invitrogen) or an RNeasy Mini kit (QIAGEN) according to the manufacturers’ instructions. A Superscript II Reverse transcriptase kit (Invitrogen) was used to make complementary DNA from 500 ng of total RNA. All qRT-PCRs were carried out in a LightCycler 480 Real-Time PCR System (Roche Applied Science). Primer sequences are given in Table S2 in Additional file 1.

### Expression microarray analysis

Total RNA was isolated from cells using TRIzol (Invitrogen). RNA integrity (RIN) was assessed using an Agilent 2100 Bioanalyzer (samples used had RIN score of ≥8.0). RNA was amplified and biotinylated using an Illumina TotalPrep RNA Amplification Kit and subsequently hybridized to Illumina mouse WG-6 Expression BeadChips. Array processing was performed at the Wellcome Trust Clinical Research Facility in Edinburgh.

Arrays were background subtracted and quantile normalized using the lumi package of the R programming language. Differential expression was determined by paired *t*-test with correction for multiple testing (Benjamini and Hohchberg).

### Luminometric methylation assay

LUMA combines differential DNA cleavage by methylation-sensitive restriction enzymes with pyrosequencing to determine genome-wide DNA methylation levels at CCGG sites and has been described in detail elsewhere [64].

### Bisulfite sequencing

Genomic DNA (500 ng) was bisulfite treated using an EZ DNA Methylation-Lightning kit (Zymo Research, Irvine, California, USA according to the manufacturer’s instructions. Bisulfite primers were designed using the ‘Bisearch’ algorithm [65] and are shown in Table S1 in Additional file 1. Products were amplified by two rounds of PCR, the second using nested primers, then resolved by agarose gel electrophoresis. A negative control reaction containing dH_2_0 in place of bisulfite converted DNA was used for each set of reactions. Products were gel extracted, cloned into pGEM T Easy vector (Promega, Fitchburg, Wisconsin, USA and transformed into Library Efficiency DH5α bacteria (Invitrogen). Blue/white screening was performed using X-gal (Sigma). Plasmid DNA from individual colonies was isolated by miniprep and sequenced using SP6 and T7 sequencing primer using the dideoxy sequencing method. The sequences were imported into the ‘BioEdit’ program [66] and aligned to a reference sequence for the region, created by *in silico* bisulfite treatment, using the ‘ClustalW’ inbuilt program. Clones with identical sequences were removed due to the possibility of PCR bias. Clones with <95% conversion rate of C to T were excluded. The methylation status of each CpG dinucleotide from each bacterial clone was assessed by the presence or absence of C to T conversion in the PCR product, and visualized using ‘QUMA’ online program [2,67] The overall percentage CpG methylation was calculated for each region.

### Data access

All data have been deposited in ArrayExpress at the European Bioinformatics Institute under the accessions E-MTAB-3172, E-MTAB-3176, E-MTAB-3177, and E-MTAB-3183 [68].

## Additional file

Additional file 1:
**A PDF document containing all supplementary data for this paper.** Included are 12 supplemental figures **(Figures S1 to S12)** and four supplemental tables **(Tables S1 to S4)**.

## Abbreviations

5hmC: 5-hydroxymethylcytosine
5mC: 5-methylcytosine
CGI: CpG island
CMS: cytosine-5-methylenesulfonate
DAPI: 4′,6-diamidino-2-phenylindole
DNMT: DNA methyltransferase
E: embryonic day
ES: embryonic stem
FCS: fetal calf serum
GEO: Gene Expression Omnibus
GLIB: glucosylation, periodate oxidation, biotinylation
GO: Gene Ontology
H3K27me3: histone H3 lysine 27 trimethylation
hMeDIP: hydroxymethylated DNA immunoprecipitation using 5hmC antibodies
IL: interleukin
IP: immunoprecipitation
LUMA: luminometric methylation assay
MACS: magnetic activated cell-sorting
MBD: methyl-binding domain
MeDIP: methylated DNA immunoprecipitation using 5mC antibodies
MEF: mouse embryonic fibroblast
PBS: phosphate-buffered saline
qPCR: quantitative polymerase chain reaction
qRT-PCR: quantitative reverse transcription polymerase chain reaction
