# Supplementary material for: Rapid reprogramming of epigenetic and transcriptional profiles in mammalian culture systems
Source: Genome Biol. 2015 Feb 4;16(1):11. doi: 10.1186/s13059-014-0576-y (PMC4334405; doi:10.1186/s13059-014-0576-y)
Supplement: Additional file 1: — A PDF document containing all supplementary data for this paper. Included are 12 supplemental figures (Figures S1 to S12) and four supplemental tables (Tables S1 to S4). [file 13059_2014_576_MOESM1_ESM.pdf]

Supplementary Figures and Tables legends

Supplementary Figure S1. Proliferation of stimulated naïve CD4⁺ T-cells in culture. (a) Histograms showing the proportions of naïve CD4⁺ T-cells that have undergone cell division 1, 3 and 5 days after stimulation with anti-CD28 and anti-CD3 as determined by carboxyfluorescein diacetate succinimidyl ester (CFSE) incorporation. The percentage of cells that have undergone at least one cell division at each time-point is shown in blue. Red-dotted line represents the CFSE profile at day one. **(b)** Bargraph showing the proportion of cells in S1/G2M phase 0, 3 and 5 days after stimulation after stimulation with anti-CD28 and anti-CD3 as determined by propridium iodide staining. **(c)** Dot-blot of a serial dilution of genomic DNA from primary MEF tissue and primary naïve CD4⁺ T-cells with an antibody specific to 5hmC. Methylene blue was used to control for loading. Control DNAs were unmodified (C), methylated (5mC) and hydroxymethylated (5hmC), respectively.

Supplementary Figure S2: Mouse embryonic fibroblasts retain their cellular identity in culture. (a) Bargraph illustrating the relative expression (arbitrary units) of two MEF-specific genes (*Lhx6* & *Lrrc15*) in 96 different mouse tissues and cell lines, identified by bioinformatics meta-analysis of published gene expression data (GEO ID: GSE1133). MEF expression is shown in red. Dotted line indicates the median expression level across all 96 tissues. **(b)** Heatmap illustrating the expression level of all 23 MEF-specific genes in all eight biological samples ($N_{CULTURE}$ = 4; N_{TISSUE} = 4). (c) Fluorescence microscopy images of MEFS during adaptation to culture stained with an antibody against Arid5b (*Arid5b*, green). The DNA was counterstained with DAPI (blue). The merged DAPI & *Arid5* image is shown on right.

Supplementary Figure S3. MeDIP-chip can detect small locus-specific, allele-specific changes in DNA methylation. (a) Promoter hypermethylation of the CpG-rich promoter of the gene *Mageh1* on the inactive X chromosome in female MEF tissue is readily detected by MeDIP-chip. Schematic representation of the 5mC profile of the promoter is shown; the figure is adapted from the UCSC Genome Browser. CpG Islands are shown in green. **(b)** Loss of methylation at the promoter of the *Xist* gene on the active X chromosome is clearly detected by MeDIP-chip. Schematic representation of the 5mC profile of the promoter is shown; the figure is adapted from the UCSC Genome Browser. **(c)** Reduced 5mC (left panel) and 5hmC (right panel) levels were observed across the female X chromosome (red dots)

suggesting genome-wide hypomethylation of the inactive X chromosome in female cells. Each data point represents the median 5mC- and 5hmC-enrichment levels as determined by MeDIP- and hMeDIP-chip in non-overlapping 2 Mb windows of the mouse genome (chromosome 14 – 19 and X).

Supplementary Figure S4. Adaptation to cell culture remodels the 5hmC profile of mouse embryonic fibroblasts. Unsupervised hierarchical clustering grouped the 5mC samples by sex, reflecting 5mC differences on the X chromosome between samples. The 5hmC profiles clustered samples by sample type (tissue v culture) reflecting the dramatic culture-induced remodelling of the 5hmC profile during adaptation to culture. Clustering was performed using the 'hclust' function in the R programming language; a distance matrix was computed for each data set using Euclidean distance, subsequently samples were clustered using *Ward's* minimum variance agglomeration method.

Supplementary Figure S5. Loss of 5hmC was highly conserved between biological replicates. (a) 5mC patterns are highly conserved between biological replicates (2 male (♂) and 2 female (♀)) of embryonic tissue and all 4 biological replicates upon adaptation (2 male (♂) and 2 female (♀)) to culture. A schematic representation of the 5mC profile of a 40 Mb autosomal region is shown; the figure is adapted from the UCSC Genome Browser. Annotated RefSeq transcripts are shown in red. Tissue profiles in blue and culture profiles in black. **(b)** 5hmC patterns are highly conserved between biological replicates of primary embryonic tissue and show clear regions of enrichment associated with transcript density. This pattern is lost in all 4 biological replicates (2 male (♂) and 2 female (♀)) upon adaptation to culture. A schematic representation of the 5hmC profile of a 40 Mb autosomal region (chromosome 14) is shown; the figure is adapted from the UCSC Genome Browser. Annotated RefSeq transcripts are shown in red. Tissue profiles in blue and culture profiles in black.

Supplementary Figure S6. The distribution of 5hmC peaks of enrichment are altered in MEFs after culture. (a) Significantly less 5hmC peak probes were detected in DNA from cultured samples. Bargraph (right panel) showing a reduction of 5hmC peak probes in DNA from cultured samples (Mann-Whitney U-test, $P < 0.01$). No reduction in peak probe number was observed for 5mC (left panel). Peak probes were defined as any five consecutive probes in which a minimum of four probes had an enrichment score above the 90th percentile. Tissue

samples are yellow and cultured samples are in purple. **(b)** Diagram illustrating the defined genomic regions that are assayed for percentage (%) 5hmC enrichment (top panel); Promoter, TSS, exon, intron, down-stream and inter-genic. On the left panel the 5mC and 5hmC distribution of all defined regions is shown. The distribution of 5hmC peaks is altered in MEFs after culture over intronic and intergenic regions. On the right panel the 5mC and 5hmC distribution of all genic regions is shown; that is intergenic and intronic probes have been excluded. Here, 5mC showed a small, but significant (Mann-Whitney U-test, $P < 0.05$) enrichment in promoter peaks in culture. 5hmC distribution over promoters and exons are also different between tissue and cultured MEF samples, showing a relative increase and decrease at promoters and exonic regions, respectively. Peak probes were defined as any five consecutive probes in which a minimum of four probes had an enrichment score above the 90th percentile.

Supplementary Figure S7. Loss of 5hmC from gene bodies in cultured MEFs is highly consistent across replicates. Schematic representation of 5hmC content across the gene body of two genes, *Vwa2* and *Klf5*, is shown for all biological replicates. 2 male (♂) and 2 female (♀) for tissue (blue profile) and cultured (black profile) MEFs. The gene organisation for *Vwa2* and *Klf5* is shown above.

Supplementary Figure S8. Agglomerative methylation in cultured MEFS of CpG island promoters across the *Hoxc (4-13)* locus. (**a**) Schematic representation of the 5mC profile across the *Hoxc* locus, showing gain of 5mC at several CpG island promoters upon adaptation of MEFs to culture. The figure is adapted from the UCSC Genome Browser. Annotated RefSeq transcripts are shown in light blue. (**b**) Bisulfite sequencing of selected *Hoxc* gene promoters in MEFs before and after culture. Filled circles (black) represent 5mCpG and open circles represent CpG. The overall amount of 5mCpG as a percentage of total CpG in each amplicon is shown under each image.

Supplementary Figure S9. Validation of hMeDIP enrichment profiles by GLIB (glucosylation, periodate oxidation, biotinylation). (**a**) Locus-specific GLIB-PCR of five candidate loci which gained (*Hoxc4, Hoxc5*), lost (*Vwa2, 1700054A03*), and retained (*Gnas*) 5hmC in culture. Tissue is in blue and cultured samples in orange. (**b**) The same candidate loci as assayed by locus-specific hMeDIP-PCR. Tissue is in blue and cultured samples in orange. The values for A and B are an average of four biological replicates, ± SD. * $P < 0.05$,

Mann-Whitney U-test. (**c**) A schematic representation of 5hmC enrichment across chromosome 17 by hMeDIP-chip (blue), and GLIB seq (purple) of primary MEFs (From *Neri et al*, 2013: Short Read Archive: SRX244234). (**d**) A schematic representation of 5hmC enrichment across the H19 locus (indicated by red bar) by hMeDIP-chip (blue), and GLIB seq (purple) (From *Neri et al*, 2013: Short Read Archive: SRX244234).

Supplementary Figure S10: hMeDIP-Seq confirms the rapid redistribution of 5hmC during adaptation of MEFs to culture. (**a**) A minimum of 30 million hMeDIP-Seq reads for each sample were uniquely mapped to the mouse genome (mm10) using the bowtie short read aligner. (**b**) hMeDIP-Seq coverage was normalised to total read count per sample (reads per million, RPM). The genomic profiles obtained by hMeDIP-Seq closely replicated those obtained by hMeDIP-chip, including (**c**) loss of 5hmC from gene bodies and (**d**) maintenance of 5hmC at the *H19* locus. (**e & f**) Gains of 5hmC were observed across all four *Hox* loci. The *Hoxc* (**e**) and *Hoxb* (**f**) loci are shown. Figures are adapted from the Integrated Genome Viewer [63].

Supplementary Figure S11: Genome-wide redistribution of 5hmC and 5mC in CD4$^+$ T-cells in culture replicates that observed in MEFs. (**a**) Clear patterning of 5hmC is lost upon adaptation of MEFs to culture. A schematic representation of the 5hmC profiles over a 40 Mb section of chromosome 14; adapted from the UCSC Genome Browser (for comparison with Figure 2h). Refseq transcripts (transcript) mapped to the region are also shown. (**b**) Bar chart showing six-fold fewer 5hmC-enriched peaks were detected in DNA from cultured samples than uncultured naïve T-cells (top panel). Bar chart showing the distribution of 5hmC peaks in genic (gene-body or promoter) and non-genic regions in non-cultured (NT) and cultured cells (Th1) CD4$^+$ T-cells. The distribution of 5hmC peaks is altered in CD4$^+$ T-cells after culture, showing a small shift towards inter-genic regions. 5hmC peaks were determined using MACS peak-finding software. Genic compartments were defined as shown in Figure S6b. (**c**) Histogram of the distribution of changes in gene body 5hmC coverage in cultured and uncultured CD4$^+$ T-cells. A marked loss of 5hmC is evident and similar to that observed in MEFs. (**d**) Histogram of the distribution of changes in promoter 5hmC coverage in cultured and uncultured CD4$^+$ T-cells. (**e**) Histogram of the distribution of changes in gene body 5mC coverage in cultured and uncultured CD4$^+$ T-cells. (**f**) Histogram of the distribution of changes in promoter 5mC coverage in cultured and uncultured CD4$^+$ T-cells.

Large changes in promoter 5mC (2-fold) in cultured are skewed towards gains, as seen in MEFs. Data was downloaded from the Gene Expression Omnibus (GEO: GSE59212)

Supplementary Figure S12: hMeDIP-qPCR confirms the generality of 5hmC redistribution during adaptation of different cell types to culture. (**a**) Bar chart of hMeDIP-qPCR showing changes in 5hmC content at six loci (3 gaining 5hmC in culture, 3 losing 5hmC in culture) in cultured MEFs. Each bar represents a single biological replicate. Error bars represent the standard deviation of multiple technical replicates. (**b**) Bar chart of hMeDIP-qPCR showing changes in 5hmC at the same six loci in cultured $CD4^+$ T-cells in the presence and absence of 1000 uM Vitamin C. Note that the scale and direction of change at each locus in MEFs and $CD4^+$ T-cells is highly similar, and Vitamin C addition results in partial-to-full rescue of 5hmC at each locus. Each bar represents a single biological replicate. Error bars represent the standard deviation of multiple technical replicates. (**c & d**) Bar chart of MeDIP-qPCR showing changes in 5mC at the same six loci in cultured MEFs (**c**) and $CD4^+$ T cells (**d**). Each bar represents a single biological replicate. Error bars represent the standard deviation of multiple technical replicates.

Supplemental Table S1. MEF-specific genes

Supplemental Table S2. Genomic features that are present on tiling microarrays.

Supplemental Table S3. Quantitative PCR (qPCR) primers.

Supplemental Table S4. GO terms associated with promoters gaining 5mC in culture

Supplementary Figure S1 (*Meehan*)

a. CSFE

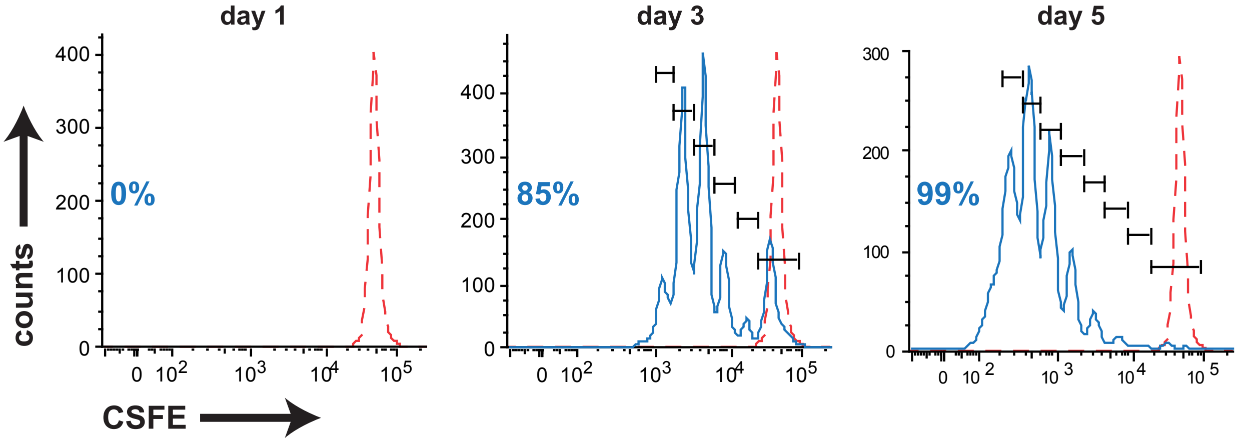

b. propridium iodide

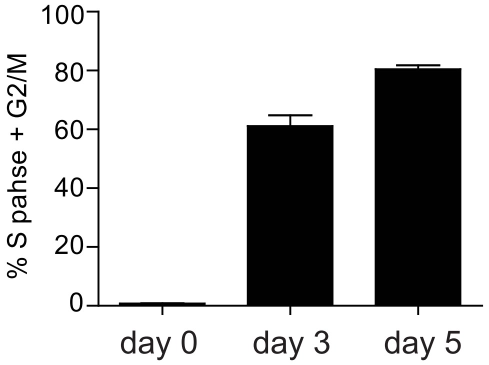

c.

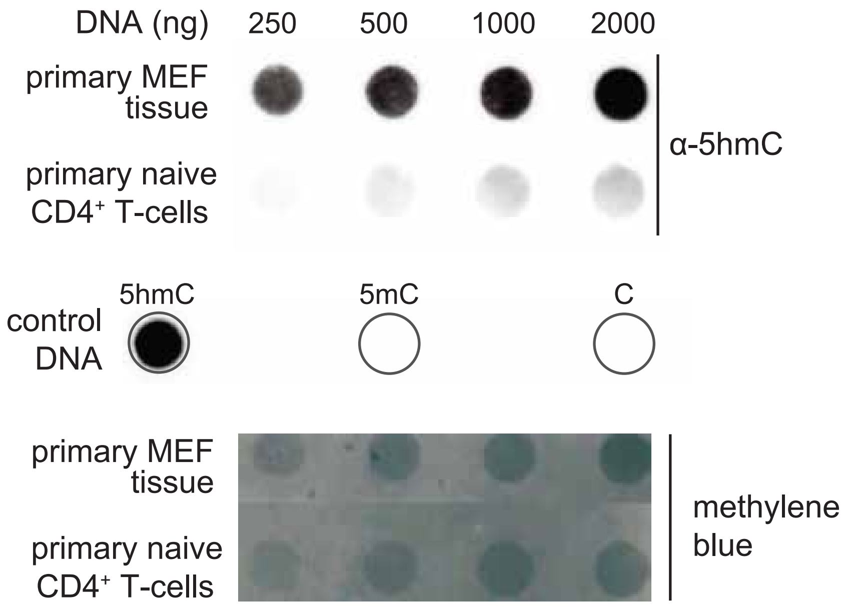

Supplementary Figure S2

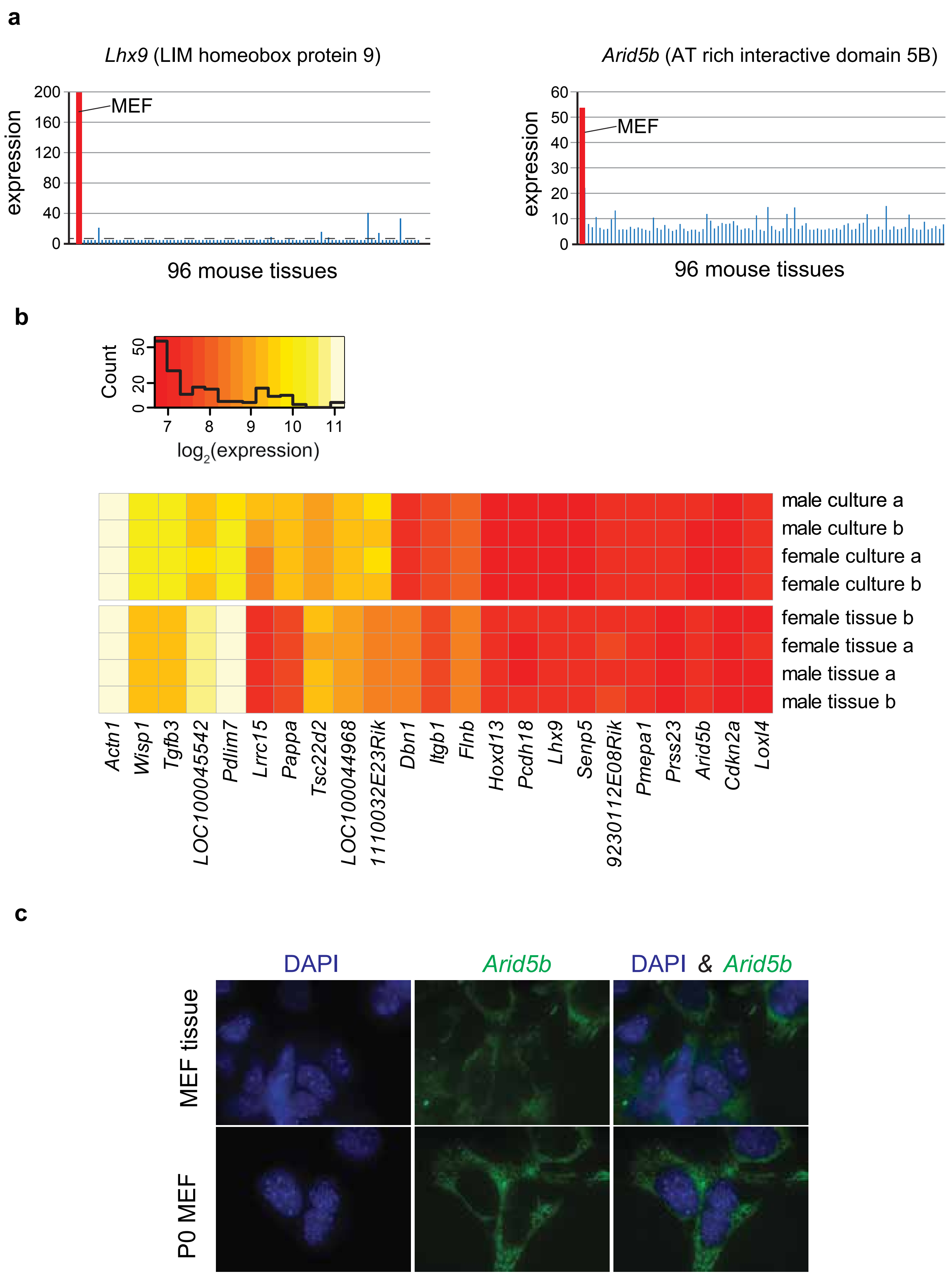

Supplementary Figure S3 (*Meehan*)

a. 5mC

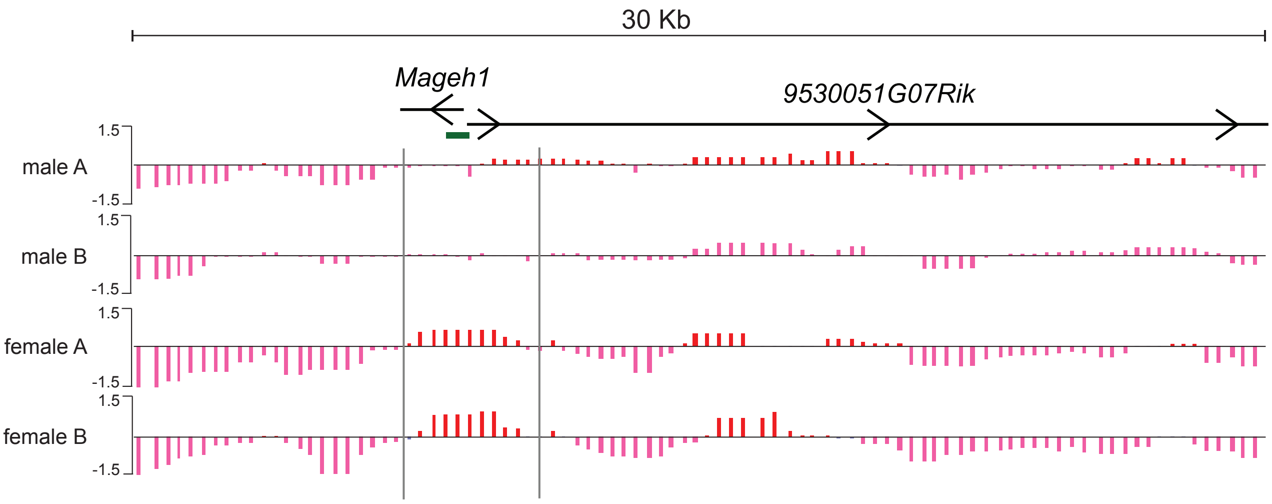

b. 5mC

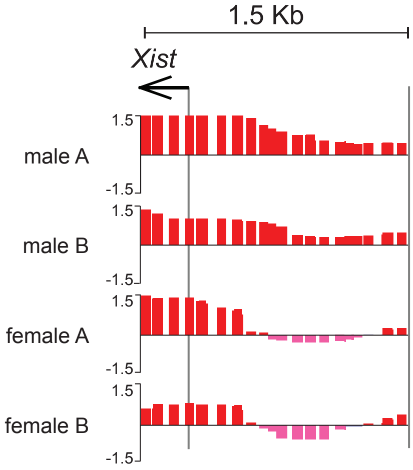

c.

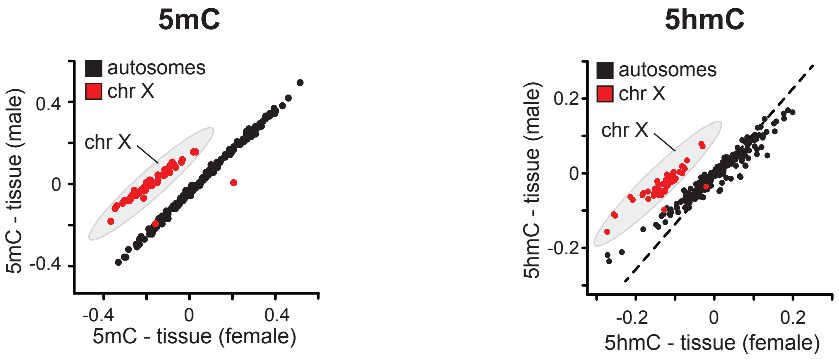

Supplementary Figure S4 (*Meehan*)

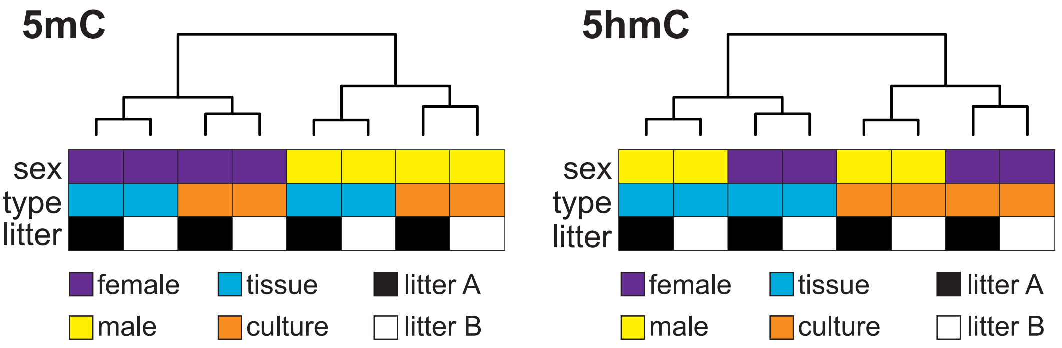

Supplementary Figure S5 (*Meehan*)

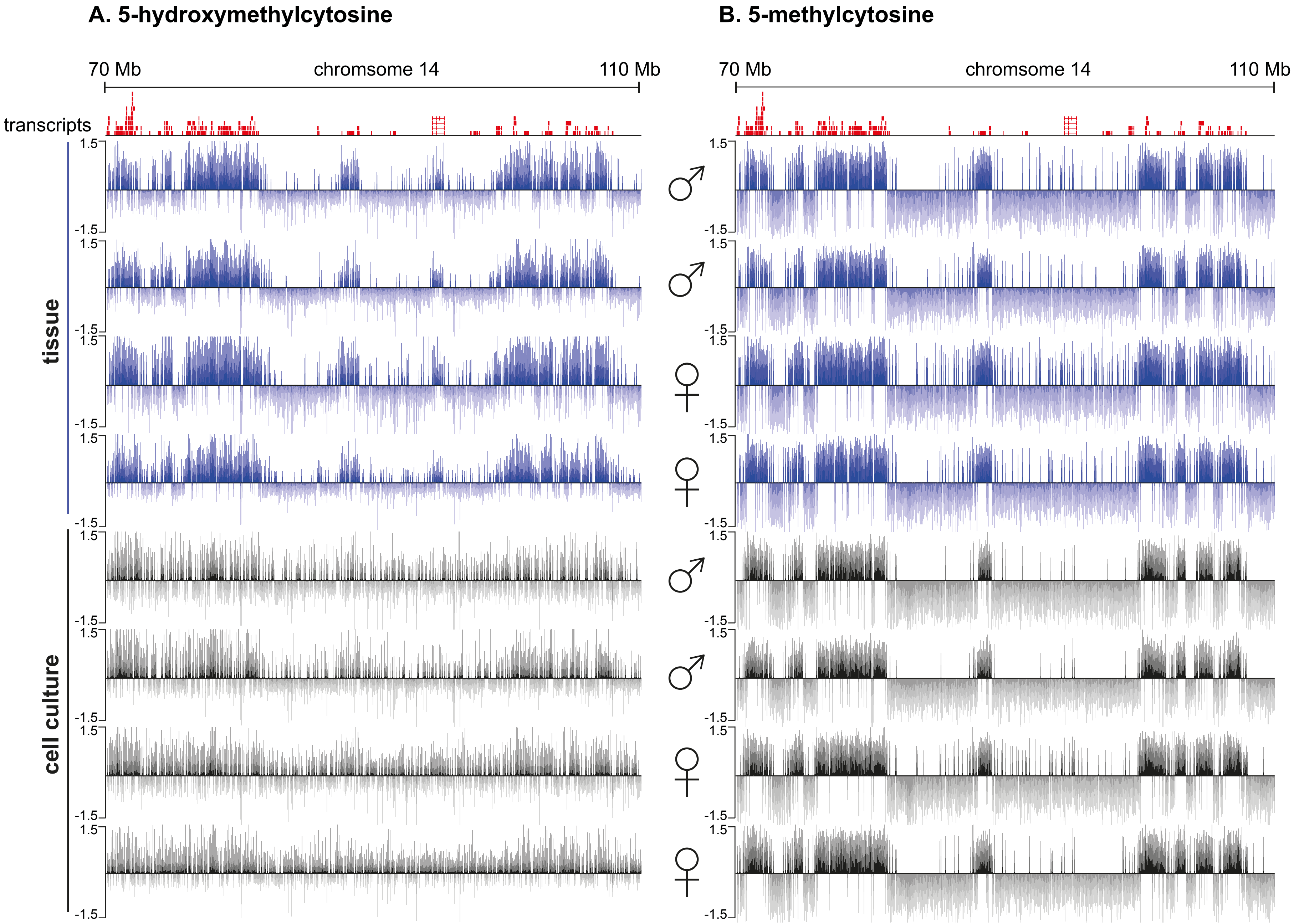

Supplementary Figure S6 (*Meehan*)

a.

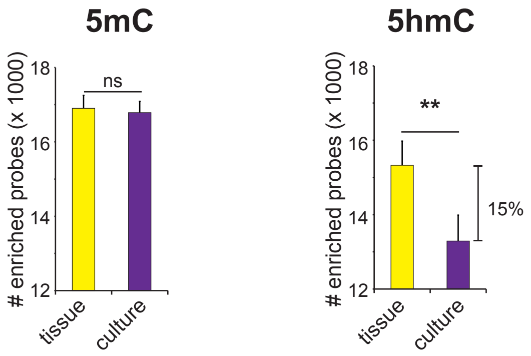

b.

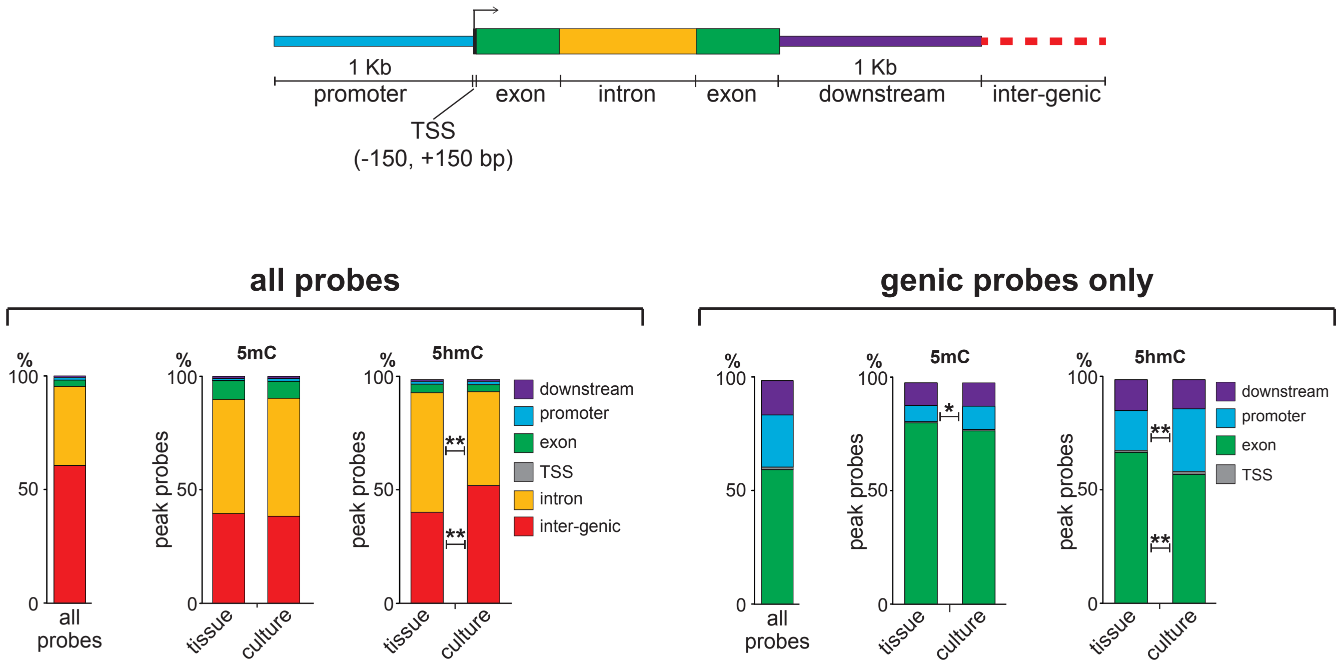

Supplementary Figure S7 (*Meehan*)

A. 5-hydroxymethylcytosine

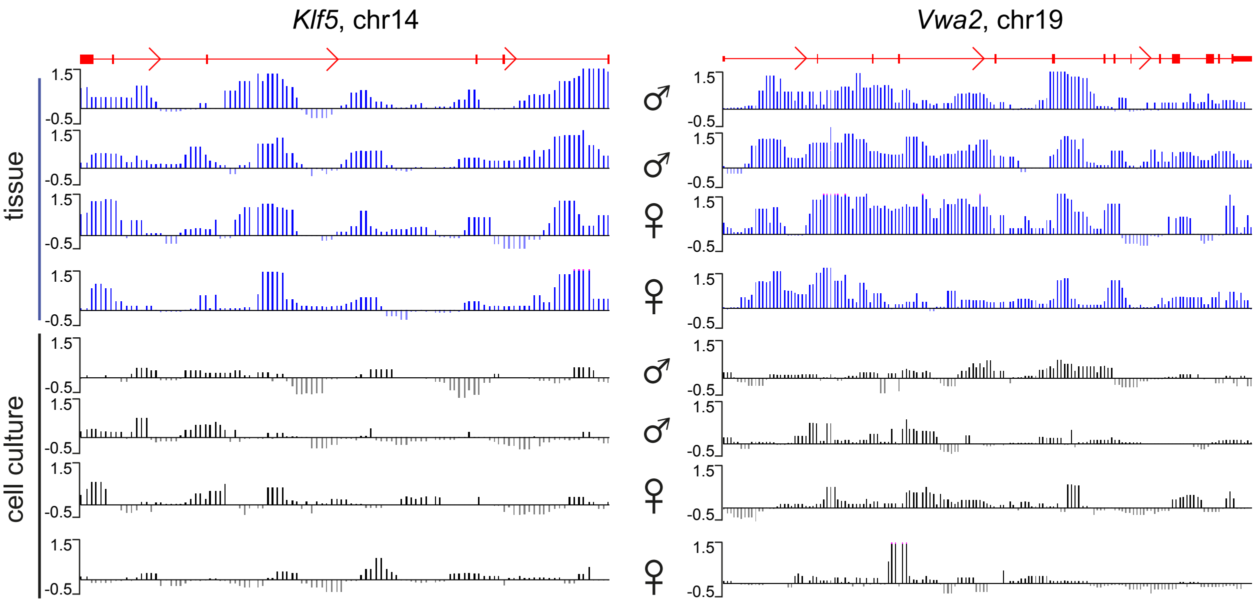

Supplementary Figure S8 (*Meehan*)

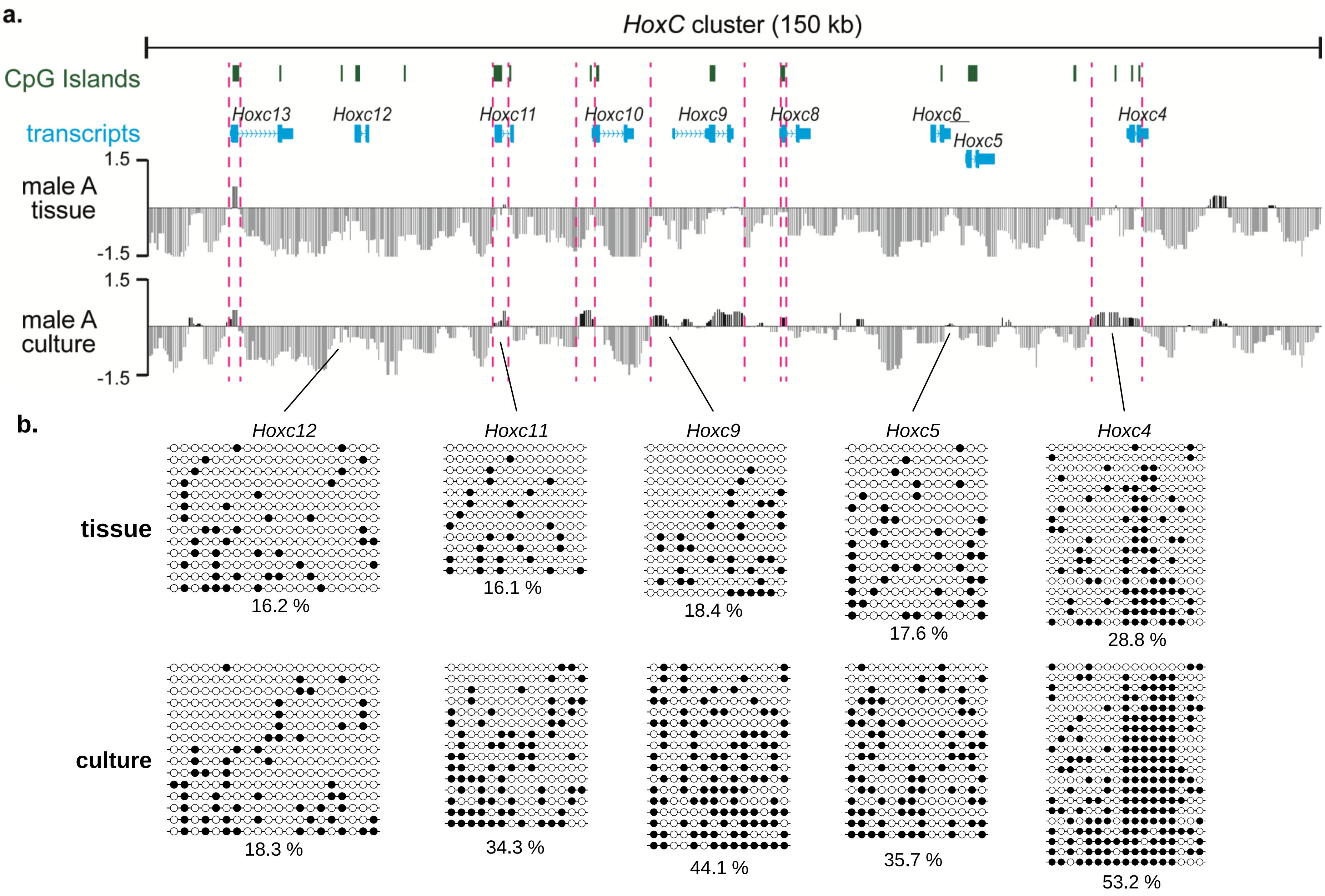

Supplementary Figure S9 (*Meehan*)

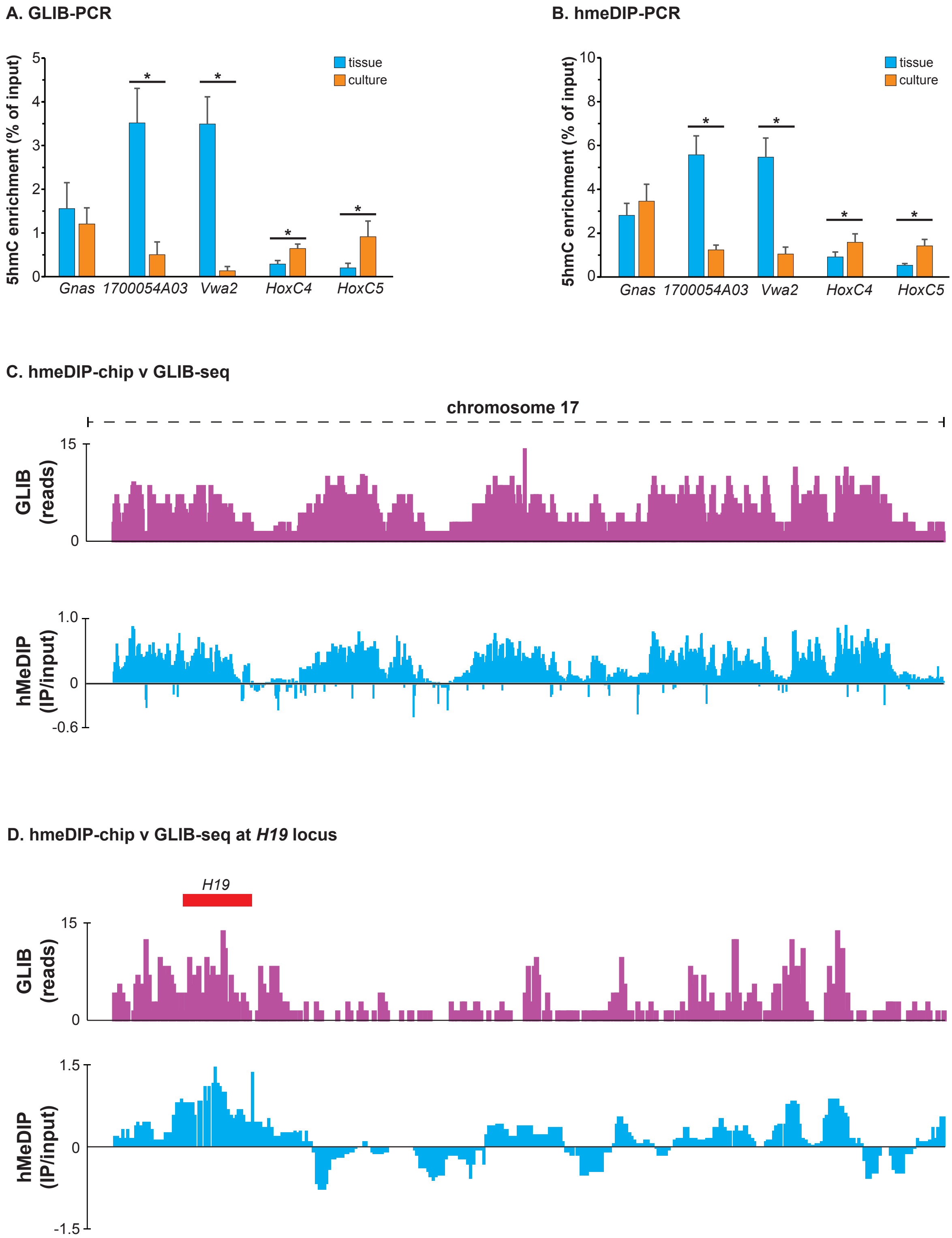

Supplementary Figure 10 (*Meehan*)

a. hMeDIP-Seq

uniquely mapped reads per sample (single end)

	tissue	culture
input	60,383,385	70,180,026
IP	30,066,313	57,412,306

b. hMeDIP-Seq v hMEDIP-chip

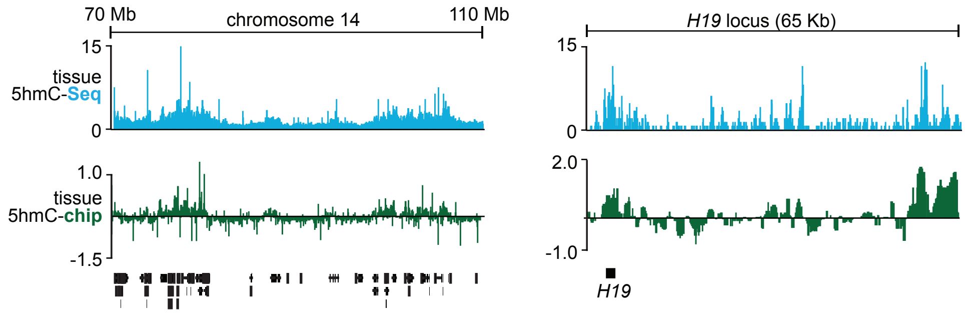

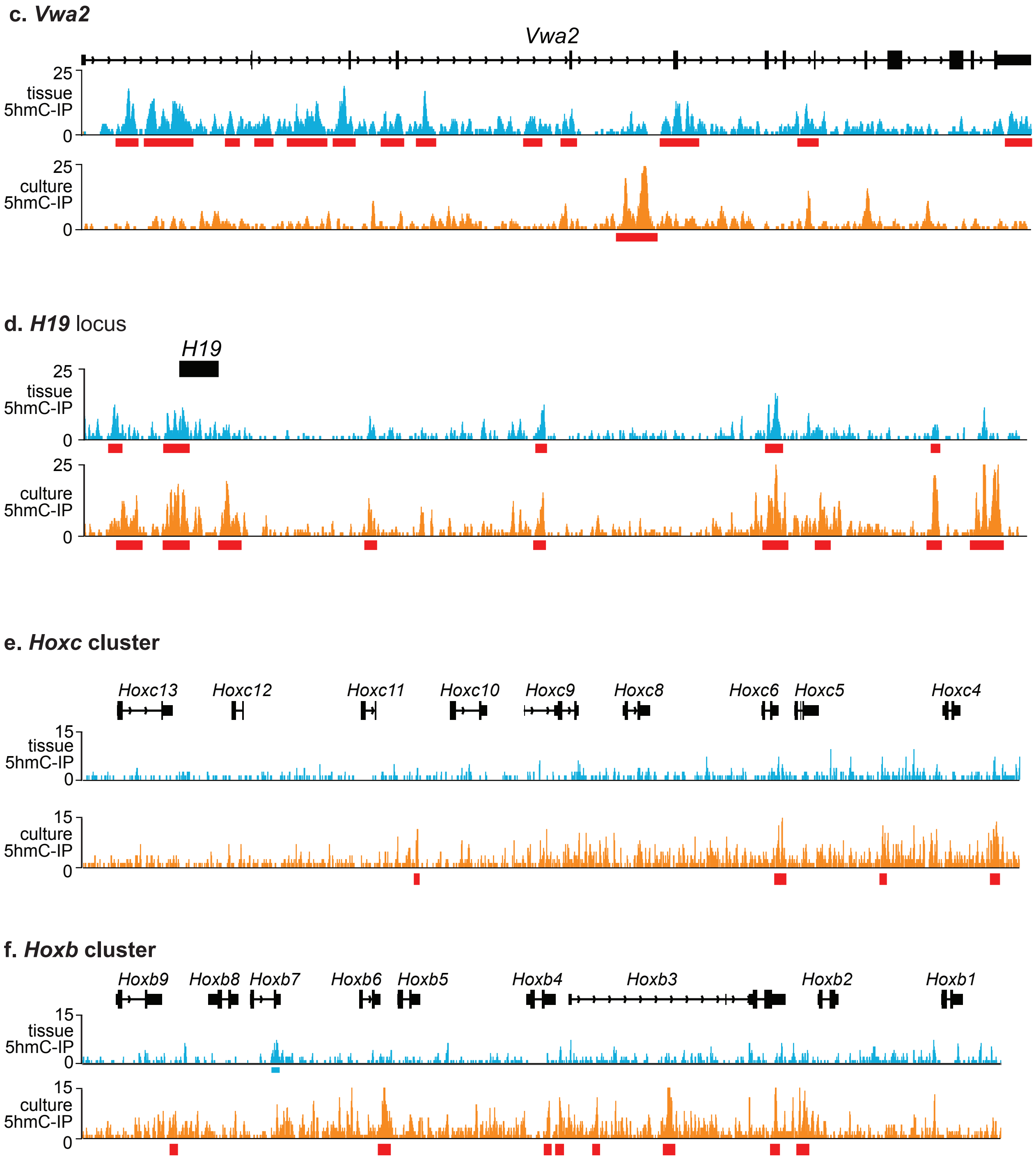

Supplementary Figure S11 (*Meehan*)

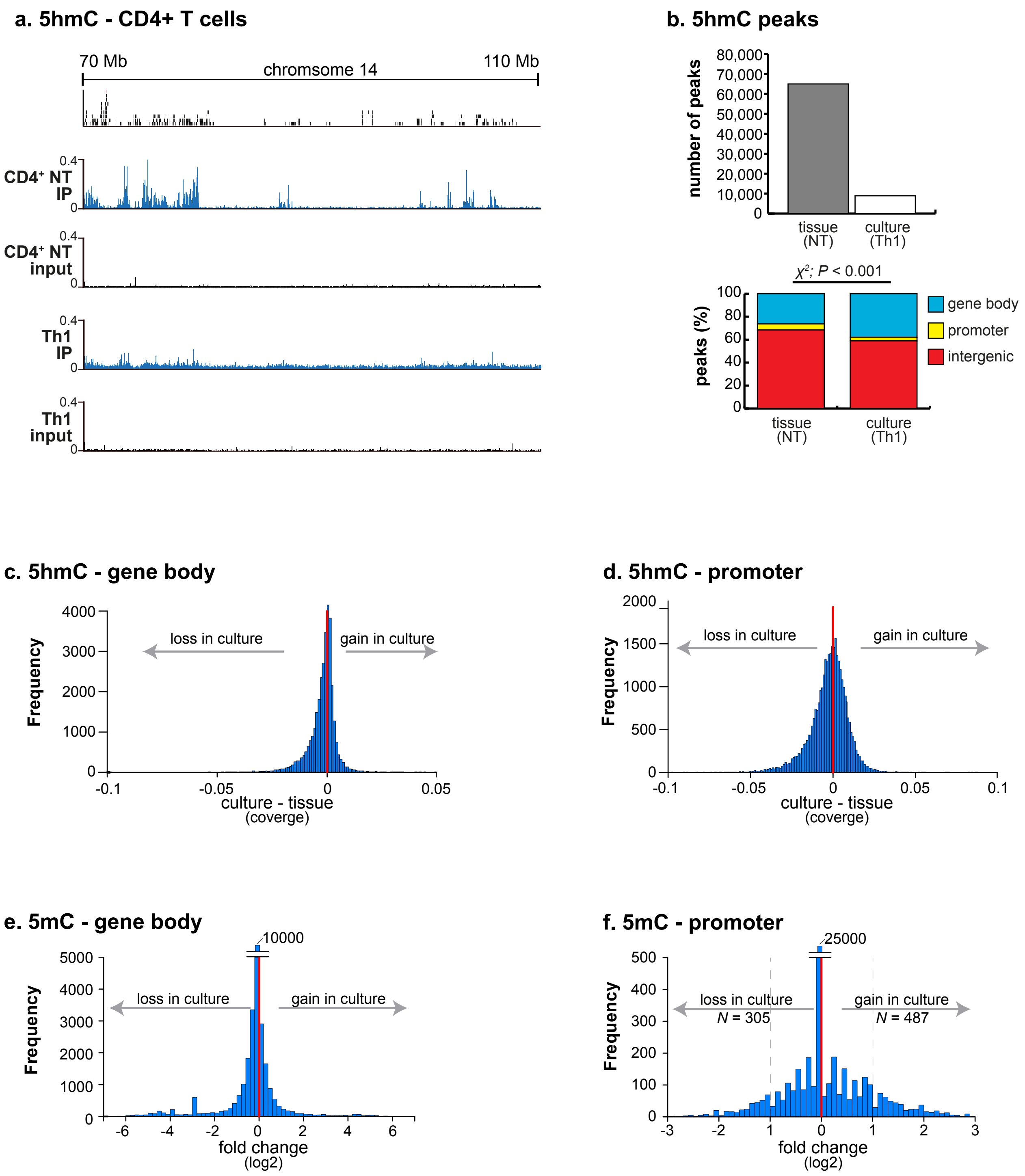

Supplementary Figure S12 (*Meehan*)

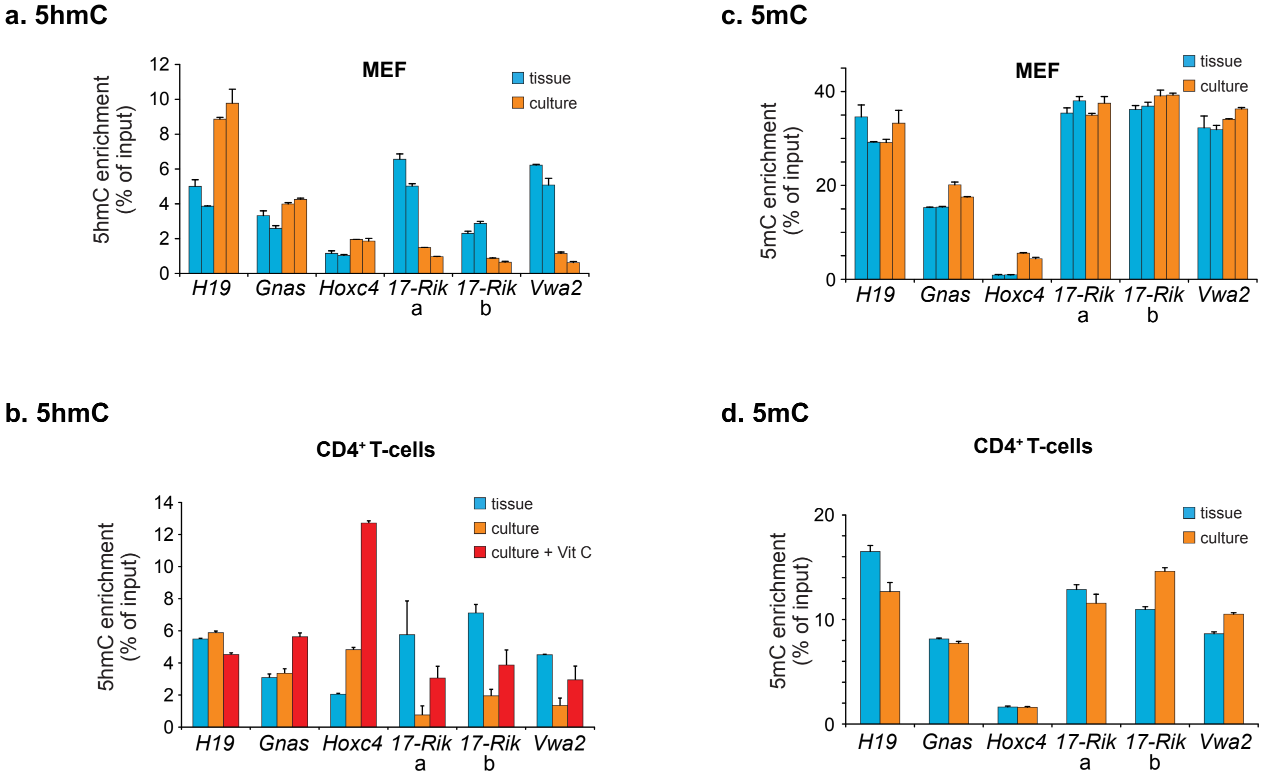

Supplemental Table S1. MEF-specific genes

Gene Symbol	Affy Probe ID	Gene Descrption
9230112E08Rik	1444156_at	RIKEN cDNA 9230112E08 gene
Actn1	1452415_at	actinin, alpha 1
Arid5b	1420972_at	AT rich interactive domain 5B (MRF1-like)
Cdkn2a	1450140_a_at	cyclin-dependent kinase inhibitor 2A
Dbn1	1451734_a_at	drebrin 1
1110032E23Rik	1429637_at	RIKEN cDNA 1110032E23 gene
Flnb	1426750_at	filamin, beta
Hoxd13	1422239_at	homeo box D13
Itgb1	1426920_x_at	integrin beta 1 (fibronectin receptor beta)
Lhx9	1429905_at	LIM homeobox protein 9
LOC100044968	1420972_at	similar to modulator recognition factor 2; AT rich interactive domain 5B (MRF1-like)
LOC100045542	1452280_at	FERM, RhoGEF (Arhgef) and pleckstrin domain protein 1 (chondrocyte-derived);
Loxl	1436063_at	lysyl oxidase-like 1
Lrrc15	1453214_at	leucine rich repeat containing 15
Pappa	1432592_at	pregnancy-associated plasma protein A
Pcdh18	1422889_at	protocadherin 18
Pdlim7	1443785_x_at	PDZ and LIM domain 7
Pmepa1	1452295_at	prostate transmembrane protein, androgen induced 1
Prss23	1446560_at	protease, serine, 23
Senp5	1440904_at	SUMO/sentrin specific peptidase 5
Tgfb3	1417455_at	transforming growth factor, beta 3
Tsc22d2	1453012_at	TSC22 domain family, member 2
Wisp1	1448593_at	WNT1 inducible signaling pathway protein 1

Supplemental Table S2. Genomic features present on tiling microarrays

chr	size (Mb)	transcripts	genes	CpG islands	genic (Mb)	non-genic (Mb)
14	107.6	266	206	240	16.4	91.2
15	100.5	1130	856	985	36.6	63.9
16	95.3	956	713	640	33.1	62.2
17	92.3	1492	1127	1114	36.3	56.0
18	87.8	722	558	699	38.2	49.6
19	58.3	974	749	787	26.6	31.7
X	163.6	1251	974	514	41.8	121.8
Y	2.9	22	21	9	1.1	1.8
Total	708.3	6813	5204	4988	230.1	478.2

Supplemental Table S3. Quantitative PCR (qPCR) primer

target name	*forward primer (5' → 3')*	*reverse primer (5' → 3')*	Length (bp)
Hoxc4 Promoter	CATCGATCCGAAATTTCCTC	TACAGCTCCTGGTGGTGATG	123
Hoxc5 Promoter	CGGCTTCCATCACTAACCTC	ATGCGCTCTTCCCAAATAAG	104
Hoxc8 Promoter	CCTATTACGACTGCCGGTTC	CGTGGTGGAAGAAGTCTTGG	123
Hoxc9 Promoter	TCAGTCTGGGCTCCAAAGTC	AGAGGTAGCCTCCCCAGAAC	130
Hoxc11 Promoter	GGCAGGAGAAGAGAACGATG	TGGGCAGATAGAGGTTGGAG	123
Hoxc12 Promoter	AAATCTGTTCCTGCCTTTCG	TCTACTCACGTGGGCGCTAC	137
*Hoxc1*3 Promoter	AGACCCAGGCTTAGCATCAC	TAAGAAATCCGGCGACTCC	93
1700054A03Rik	TTGATCAGGTTTCCCAAAGC	TCTCTGCCTTTGTTTCTCTGG	119
1700054A03Rik 1st intron	GGCTCAGCCTGATTCCAC	CAGTCGGTCGAAATCTAAAGG	112
1700054A03Rik 2nd intron	GACTCTGGTTGTCCGATTCC	TAGCACAATGGAGCAAGCAC	112
Igsf5 promoter	ATGTCACAGGATCCCAGTCC	TGCTAGGTGGTCAGGGAAAG	118
Klf5 intron	CCTTGGTAAATCGTCGTTGC	TGAAGCCACTGGTGAAACTG	115
Lrrc30 5’ UTR	TGCCTCAGTTGTGCTTTTTG	GCTGCACATTTCCCATAATC	107
Lrcc30 1st exon	AGCTGGGATAGGCAGTTGTG	AGAGCCTCAAGGTCCTGTTC	115
Vwa2 1st intron	GGAAGCCTTACGCTGTTATCC	CTTAAGCTGGGCAGGAACTG	114
Vwa2 1st intron	CTGGGCCTGAGCAAGATATAG	AGCCAATGTGAGAACAGGAAG	125
Vwa2 C 1st intron	GTCTCCCTTTTCCCGAACTC	GACACAGCAAGGAACAAAAGC	124
H19 genic	CCAGCAGCTCCCCTTTATC	GGAAAACATCGGAGTGAAGC	126
H19 genic/promoter	AGAATTCCCGCCCCTACTC	GGAGTTGTGGTGAGGCTGTC	157
H19 inter-genic A	CGGACACCTCCAATTTGGTC	CAACCACCACAAGGTGACAG	90
H19 inter-genic B	TCACTTCCATCCACCATGC	GTGTCGAAGCCTGCAAAAG	128
Gnas downstream	AGGATGGAGGCTCAATATGC	GATAGCATGTCACCGGACTG	101

Supplemental Table S4. GO terms associated with promoters gaining 5mC in culture.

Cluster 1, enrichment score: **1.6**

**GO terms**		**# genes**	**unadjusted *P*-value**
pattern specification process		10	0.01
regionalization		8	0.03
anterior/posterior pattern formation		7	0.04

Cluster 2, enrichment score: **1.26**

**GO terms**		**# genes**	**unadjusted *P*-value**
muscle organ development		8	0.01
muscle cell development		4	0.01
striated muscle cell development		3	0.06
muscle fiber development		3	0.06
striated muscle cell differentiation		4	0.06
muscle tissue development		5	0.08
muscle cell differentiation		4	0.13
striated muscle tissue development		4	0.16
cardiac muscle tissue development		3	0.21

Cluster 3, enrichment score: **1.24**

**GO terms**		**# genes**	**unadjusted *P*-value**
gut morphogenesis		3	0.04
digestive tract morphogenesis		3	0.06
digestive system development		3	0.06
gut development		3	0.09

Cluster 4, enrichment score: **1.16**

**GO terms**		**# genes**	**unadjusted *P*-value**
genitalia development		4	0.01
sex differentiation		6	0.03
reproductive developmental process		7	0.12
urogenital system development		4	0.25
development of primary sexual characteristics		3	0.41
